# Biopesticide Compounds from an Endolichenic Fungus *Xylaria* sp. Isolated from the Lichen *Hypogymnia tubulosa*

**DOI:** 10.3390/molecules30030470

**Published:** 2025-01-22

**Authors:** Fotios A. Lyssaios, Azucena González-Coloma, María Fe Andrés, Carmen E. Díaz

**Affiliations:** 1 Instituto de Productos Naturales y Agrobiología, CSIC, Avda. Astrofísico F. Sánchez 3, 38206 La Laguna, Tenerife, Spain; f.lyssaios@ipna.csic.es; 2 Instituto de Ciencias Agrarias, CSIC, Serrano 115-dpdo, 28006 Madrid, Spain; mafay@ica.csic.es

**Keywords:** *Xylaria* sp., endolichenic fungi, (+)-9-hydroxypiliformic acid, (+)-8-hydroxypiliformic acid, biopesticide activity

## Abstract

Endolichenic fungi represent an important ecological group of microorganisms that form associations with photobionts in the lichen thallus. These endofungi that live in and coevolve with lichens are known for synthesizing secondary metabolites with novel structures and diverse chemical skeletons making them an unexplored microbial community of great interest. As part of our search for new phytoprotectants, in this work, we studied the endolichenic fungus *Xylaria* sp. isolated from the lichen *Hypogymnia tubulosa*, which grows as an epiphyte on the bark of the endemic Canarian tree *Pinus canariensis*. From the extract of the liquid fermentation, we isolated two unreported piliformic derivatives, (+)-9-hydroxypiliformic acid (**1**) and (+)-8-hydroxypiliformic acid (**2**), along with four previously reported compounds, (+)-piliformic acid (**3**), hexylaconitic acid A anhydride (**4**), 2-hydroxyphenylacetic acid (**5**), and 4-hydroxyphenylacetic acid (**6**). Their structures were elucidated based on NMR and HRESIMS data. The extract and the isolated compounds were tested for their insect antifeedant (*Myzus persicae*, *Rhopalosiphum padi*, and *Spodoptera littoralis*), antifungal (*Alternaria alternata*, *Botrytis cinerea*, and *Fusarium oxysporum*), nematicidal (*Meloidogyne javanica*), and phytotoxic effects on mono- and dicotyledonous plant models (*Lolium perenne* and *Lactuca sativa*). Compounds **4**, **5**, and **6** were effective antifeedants against *M. persicae* and **4** was also active against *R. padi*. Moreover, **3** and **4** showed antifungal activity against *B. cinerea* and **4** was the only nematicidal. The extract had a strong phytotoxic effect on *L. sativa* and *L. perenne* growth, with compounds **3**, **4**, and **5** identified as the phytotoxic agents, while at low concentrations compounds **3** and **4** stimulated *L. sativa* root growth.

## 1. Introduction

Endolichenic fungi represent an important ecological group of microorganisms that form associations with photobionts in the lichen thalli [1]. These fungi live symbiotically inside the lichen, similar to how endophytic fungi inhabit plant tissues [2]. In this symbiosis, the lichen provides the living environment and nutrients for the survival of the endolichenic fungi and in turn, they produce a variety of secondary metabolites that help the lichen to accelerate its growth and to protect their host during biotic and abiotic stress. The potential of endolichenic fungi to produce novel structures, diverse skeletons, and extensive bioactivities has gained significant research attention due to their possible applications in medicine and agriculture [3].

Fungi from the Xylariaceae family are frequent saprotrophs and have been described as endophytes of phylogenetically diverse plant and lichen species from a variety of ecosystems [4,5,6,7,8]. Furthermore, xylarialean endophytes can be symbiotic or saprotrophic [9]. Endolichenic isolates from the *Xylaria* genus have demonstrated the ability to produce a great diversity of secondary metabolites with a wide range of activities. Several novel antimicrobial eremophilane sesquiterpenes, such as eremoxylarins D-J, have been isolated from *X. hypoxylon* from the lichen *Rhizocarpon geographicum* [10]. Additionally, the nematicidal polyketide grammicin was isolated from *X. grammica* associated with the lichen *Menegazzia* sp. [11]. A variety of compounds, including aplysinopsin, ophiocerin B, and the polyketides piliformic acid and methyl xylariate C, were isolated from *X. venustula*, associated with the lichen *Usnea baileyi* [6]. Furthermore, endolichenic *Xylaria* strains have also been reported to produce novel cyclic peptides, including the cyclic depsipeptide xylaroamide A from *Xylaria* sp. associated with *Usnea* sp. [12]. Also, a new nitrogen-containing phenolic compound with cytotoxic activity has been discovered from *X. psidii* [13].

Secondary metabolites produced by *Xylaria* species have also shown phytoprotectant activities, including herbicidal, fungicidal, and insecticidal [14]. Various endophytic isolates of *Xylaria* sp. have been reported to produce griseofulvin [15,16,17], a metabolite that inhibits the growth of several species of fungal plant pathogens [17]. *Xylaria* sp., isolated from *Vitis labrusca*, produces diplosporin [18], a compound toxic to the polyphagous insect *Spodoptera frugiperda* when added to an artificial diet [19]. Additionally, cytochalasin E isolated from the endophytic fungus *Xylaria* sp. showed phytotoxic effects on *Lactuca sativa* and *Raphanus sativus* seedlings [20].

The excessive use of synthetic pesticides needed to increase crop production and productivity [21] has led to pathogenic resistance, and environmental problems affecting soil biodiversity, beneficial insects, aquatic life, and human health [22]. Biopesticides of natural origin are an alternative to protect plants with lower environmental impact [23]. In this work, the endolichenic fungal strain HYP6 isolated from the lichen *Hypogymnia tubulosa* was fermented in a malt liquid medium and extracted with ethyl acetate (EtOAc). The fractionation of the extract gave two unreported compounds and four known metabolites that have been identified based on their spectroscopic data. The extract and pure compounds were tested for their insect antifeedant (*Myzus persicae, Rhopalosiphum padi*, and *Spodoptera littoralis*), nematicidal (*Meloidogyne javanica*), fungicidal (*Alternaria alternata*, *Botrytis cinerea*, and *Fusarium oxysporum*), and phytotoxic effects on mono- and dicotyledonous plant models (*Lolium perenne* and *Lactuca sativa*).

## 2. Results and Discussion

### 2.1. Fungal Identification

**Figure 1:**
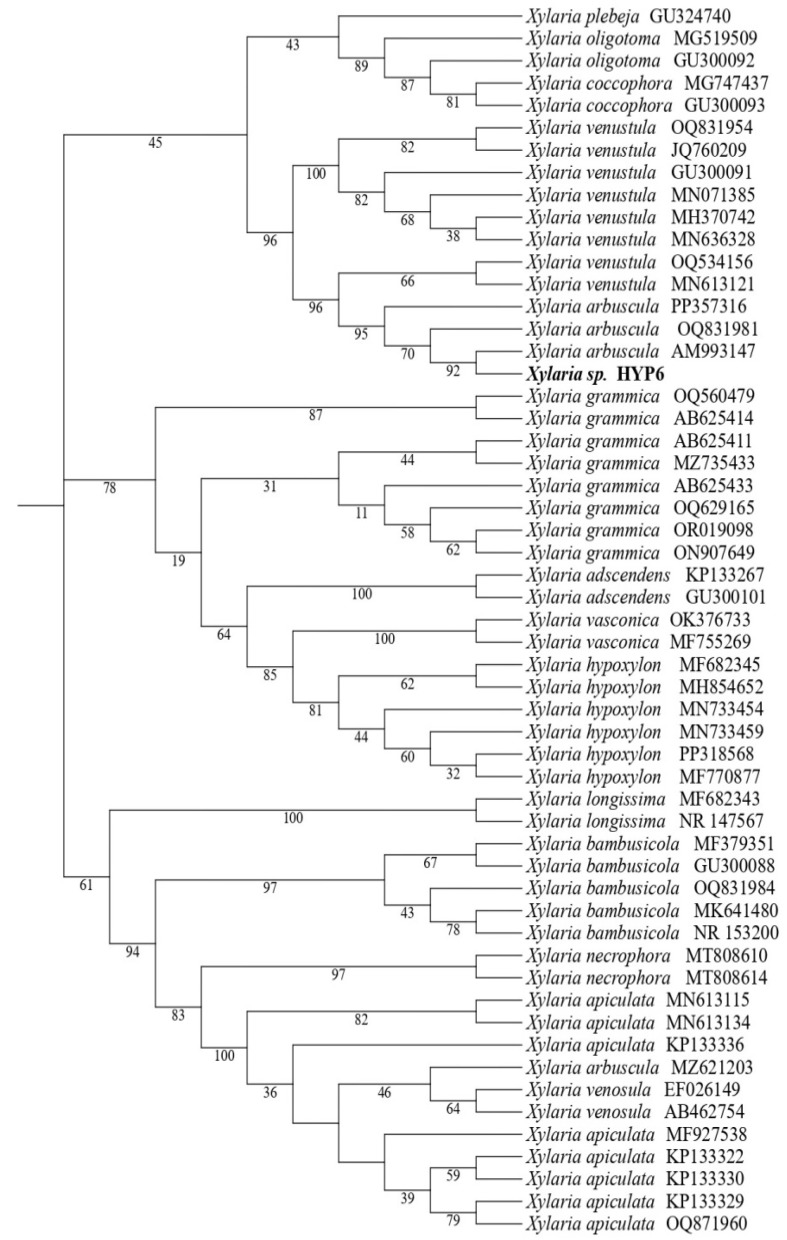
Phylogenetic maximum likelihood tree (T92+G) of *Xylaria* sp. (HYP6) based on the selected ITS rDNA sequences obtained from GenBank. Confidence values from a 5000-replicate bootstrap analysis are shown at each branch node.

The endolichenic fungal strain HYP6 was isolated from the lichen *Hypogymnia tubulosa,* growing on the bark of *Pinus canariensis*, an endemic tree of Tenerife in the Canary Islands (Spain). The rDNA ITS was sequenced and compared with those deposited in the NCBI-Genbank. To refine the taxonomic assignment, a total of 55 related sequences were used to conduct maximum likelihood phylogenetic analysis using the Tamure-3 parameter (T92+G) model and a bootstrap test with 5000 runs. The results indicated that strain HYP6 is closely related to *Xylaria arbuscula* (Figure 1). Based on multigene phylogenetic analyses [24], *X. arbuscula* is actually considered a species complex, which predominantly includes species from the tropical *Xylaria* group [25]. According to these studies and the results described above, the fungal strain HYP6 was identified as *Xylaria* sp. within the *X. arbuscula* complex.

### 2.2. Structure Elucidation

**Figure 2:**
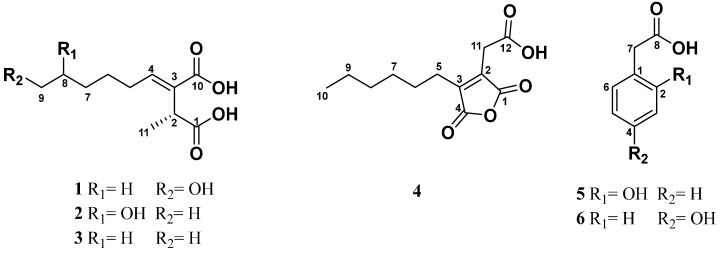
Compounds of endolichenic *Xylaria* sp. (HYP6).

The fractionation of the extract from the culture broth of strain HYP6 fermented in the malt liquid medium led to the isolation and structure elucidation of two unreported piliformic derivatives, (+)-9-hydroxypiliformic acid (**1**) and (+)-8-hydroxypiliformic acid (**2**), together with the previously described (+)-piliformic acid (**3**), the major metabolite of the extract; hexylaconitic acid A anhydride (**4**); 2-hydroxyphenylacetic acid (**5**); and 4-hydroxyphenylacetic acid (**6**) (Figure 2).

**Figure 3:**
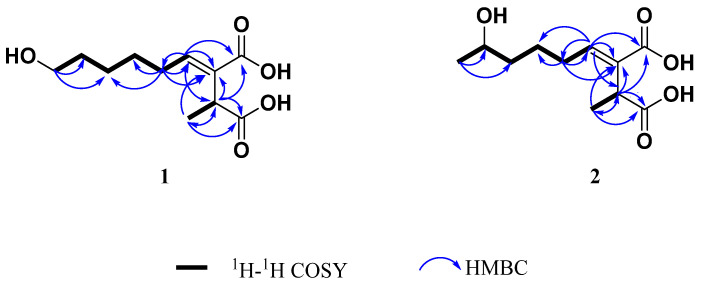
Key ^1^H-^1^H COSY and HMBC correlations of **1** and **2**.

Compound **1** was isolated from a colorless oil. Its molecular formula was determined as C_11_H_18_O_5_ by (+)-HRESIMS at *m*/*z* 253.1059 [M + Na]^+^ (calcd. for C_11_H_18_O_5_Na, 253.1052), indicating three degrees of unsaturation. The IR spectrum showed absorption bands at v_max_ 3355 (OH), 1689 (conjugated COOH), and 1637 (C=C) cm^−1^ (Figure S8). The ^1^H NMR spectrum (Table 1 and Figure S1) displayed signals of one methyl doublet at δ_H_ 1.31 (d, *J* = 7.1 Hz, H-11) coupled with the signal of a proton at δ_H_ 3.63 (dq, *J* = 7.1 Hz, H-2), and one olefinic proton at δ_H_ 6.82 (t, *J* = 7.6 Hz). Other signals observed were multiplets attributed to the protons of three overlapped methylenes of an aliphatic chain at δ_H_ 1.42–1.66 and a signal at δ_H_ 3.55 (t, *J* = 6.6 Hz, H-9), which was consistent with the presence of a hydroxymethylene group in the molecule. Analysis of the ^13^C NMR (Table 1 and Figure S2) data, in combination with the gHSQC spectrum (Figure S4), revealed the presence of eleven carbon signals, which could be assigned to two carbonyl groups at δ_C_ 178.0 (C-1) and 170.3 (C-10), two olefinic carbons at δ_C_ 134.5 (C-3) and 144.7 (C-4) of a trisubstituted double bond, one oxygenated methylene sp^3^ at δ_C_ 62.8 (C-9), one methine sp^3^ at δ_C_ 39.0 (C-2), one methyl at δ_C_ 16.4 (C-11), and four methylene sp^3^ carbons at δ_C_ 29.4 (C-5), 29.6 (C-6), 26.7 (C-7), and 33.4 (C-8). The ^1^H-^1^H COSY NMR spectrum of **1** (Figure 3 and Figure S3) showed coupling between the H-2 and H-11, and also established a partial structure from the olefinic proton at δ 6.85 (H-4) along with an aliphatic chain with a spin system of the methylene protons H-5/H-6/H-7/H-8/H-9.

The HMBC spectrum displayed correlations from H-2 to C-1, C-3, C-4, C-10, and C-11, as well as from H_3_-11 to C-1, C-2, and C-3, indicating that the two carboxyl groups are on C-2 and C-3. The double bond between C-3 and C-4 was evidenced by HMBC correlations from H-2 to C-4 and H-4 to C-2, C-6; and C-10. (Figure 3). Moreover, the presence of a hydroxyl group at the C-9 position was supported by the HMBC correlations from H-9 to C-7 and C-8 (Figure S5). Therefore, the planar structure of **1** was determined as shown in Figure 3. All the above-mentioned data are very similar to the fungal metabolite piliformic acid (**3**), also isolated from this fungal strain [26,27]. The main difference is the absence of a terminal methyl group at the side chain and the presence of a hydroxymethylene group at C-9 in compound **1**.

The NOESY correlation observed between H-2 and H-5 established the (*E*) configuration of the double bond. Compound **1** is also dextrorotatory like (+) piliformic acid (**3**) [28] obtained by chemical synthesis. The ECD spectrum of **1** (Figure S23) showed a positive Cotton effect at 230 nm, with an opposite sign to that shown by Xylaril acid A, a piliformic derivative whose structure and absolute configuration at C-2 (2R) and have been determined by X-ray diffraction [29], which suggested an absolute 2S configuration at C-2 for compound **1**. Therefore, the structure of **1** was established as (+)-(*E*)-(2S)-9-hydroxypiliformic acid.

Compound **2** exhibited HRMS (ESI+) *m/z* 253.1058 [M + Na]^+^, indicating its molecular formula C_11_H_18_O_5_Na, which is identical to **1**. The ^1^H NMR spectrum (Table 1 and Figure S9) showed similar signals to those observed for **1**, except for the presence of a sextet of a hydroxymethine signal at δ_H_ 3.74 (*J* = 6.1 Hz) and a methyl doublet at δ_H_ 1.16 (*J* = 6.1 Hz) instead of a triplet at δ_H_ 3.55 in **1**. The ^13^C NMR spectrum displayed the signals of an additional methyl carbon at δ_C_ 23.5 (C-9) and an oxymethine sp^3^ at δ_C_ 68.2 (C-8) (Figure S9)**.** The hydroxyl group is located on C-8, as supported by the ^1^H-^1^H COSY correlation of H-9 (δ 1.16)/H-8 (δ 3.74) together with the HMBC correlations of H-9 to C-7 and C-8 (Figure 3 and Figure S13). Comparison of the CD data with those **1** revealed similar Cotton effects (Figure S23), indicating the configuration at C-2 as S. A detailed analysis of the ^13^C NMR (Table 1 and Figure S9) showed double signals for C-5, C-6, C-7, and C-8, suggesting that compound **2** was the mixture of two epimers at C-8 and mixed with the ratio about 2:1 according to the height of double peaks. These observations, together with a careful analysis of the COSY, HMBC, and NOESY experiments, led to the identification of the structure of **2** as a mixture of two epimers of (+)-(*E*)-(2S)-8-hydroxypiliformic acid

Compound **4** was identified as hexylaconitic acid A anhydride, which was first isolated from *Aspergillus niger* [30] and later obtained by chemical synthesis [31,32]. In this work, we have completed the assignments of the proton and carbon resonances in the ^1^H and ^13^C spectra (Table S1) using 2D-NMR experiments (COSY, HSQC, and HMBC).

Other known compounds isolated from this fungus were identified as (+)-piliformic acid (**3**) [26,27,28], previously isolated from different fungi belonging to the *Xylaria* genus [33], 2-hydroxyphenylacetic acid (**5**) [34], and 4-hydroxyphenylacetic acid (**6**) [35].

### 2.3. Phytoprotectant Activity

The EtOAc extract and the isolated compounds, except compound **1** due to its low yield, were tested for their phytoprotectant effects against insect pests (*Myzus persicae*, *Rhopalosiphum padi*, and *Spodoptera littoralis*), the plant parasitic nematode *Meloidogyne javanica* and fungal phytopathogens (*Alternaria alternata*, *Botrytis cinerea*, and *Fusarium oxysporum*). The extract showed strong antifeedant effects against *M. persicae,* followed by moderate activity against *R. padi*. This extract showed a potent fungal activity against *B. cinerea,* and was moderately active on *F. oxysporum* and *A. alternata* (Table 2).

Among the compounds tested on insect pests (Table 2), **4** showed strong antifeedant activity against *M. persicae* (EC_50_ value of 1.6 µg/cm^2^, five times over the positive control thymol) and *R. padi* (EC_50_ value of 8.9 µg/cm^2^, two times over thymol). Additionally, **5** (EC_50_ of 4.5 µg/cm^2^, two times over thymol) and **6** (EC_50_ value of 15.5 µg/cm^2^, two times below thymol) showed selective antifeedant activity against *M. persicae*. None of the tested compounds showed significant antifeedant activity against *S. littoralis*. Compound **4** also exhibited a strong inhibition of mycelial growth of *B. cinerea* (EC_50_ value of 0.12 mg/mL, 22 times over thymol) and moderate activity against *A. alternata* (EC_50_ value of 0.24 mg/mL, 5 times over thymol), whereas **3** was moderately active against *B. cinerea* (EC_50_ value of 0.36 mg/mL, 7 times over thymol). Compound **4** was very active against *M. javanica* (LD_50_ value of 0.10 mg/mL, similar to thymol).

**Figure 4:**
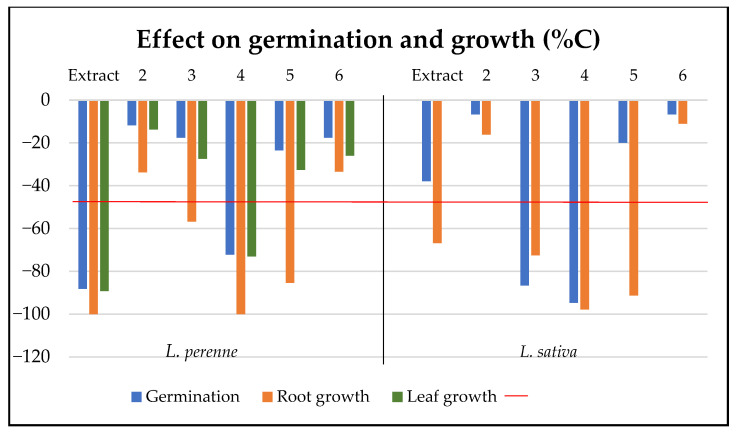
Phytotoxic effects of the extract (EtOAc, 0.4 mg/mL) and compounds (**2**–**6**, 0.2 mg/mL) on *Lolium perenne* and *Lactuca sativa*. Bars longer than the −50% threshold (red line) are significantly different from the control (*p* < 0.05, Mann–Whitney U test).

The extract and compounds were also tested for phytotoxic effects on seeds of mono- and dicotyledonous plant species (*Lolium perenne* and *Lactuca sativa*) (Figure 4). The extract strongly inhibited the germination of *L. perenne* (88.2% inhibition after 7 days). The extract also exhibited phytotoxic effects on *L. perenne* (89.3% and 100% inhibition of leaf and root growth, respectively) and *L. sativa* (66.8% inhibition of root growth). Among the compounds tested, **3** affected the germination of *L. sativa* (86.7% inhibition) and decreased the root growth of *L. perenne* (56.8% inhibition) and *L. sativa* (72.6% inhibition). Compound **4** significantly affected both species (*L. perenne* and *L. sativa*)*,* inhibiting germination (72.2% and 94.7% inhibition, respectively) and growth (100% root and 73.1% leaf growth inhibition *L. perenne*). Compound **5**, with reported phytotoxic effects [36,37], inhibited the root growth of *L. perenne* and *L. sativa* (85.4% and 91.3% inhibition, respectively) (Figure 4). The most phytotoxic compounds (**3** and **4**) were further tested in dose–response experiments against the two plant species. At lower concentrations, both compounds stimulated the root growth of *L. sativa.* Compound **3** increased the root length up to 170% at a concentration of 0.1 mg/mL while **4** caused an increase up to 200% at 0.05 mg/mL (Figure S24).

Compound **3** showed a moderate antifungal effect against *B. cinerea*. Previous reports showed moderate activity of **3** against *Colletotrichum gloeosporioides*, one of the phytopathogenic fungi responsible for the anthracnose disease [38]. Compound **4**, with aphid antifeedant, antifungal, and nematicidal effects has been previously reported for its fungicidal activity against *Neurospora crassa* [39], but was inactive against other plant pathogenic fungi such as *Gaeumannomyces graminis* var. *tritici*, *Rhizoctonia solani*, and *Phytophthora cinnamomic* [40]. This may indicate a species-dependent antifungal effect. However, this is the first report on the aphid antifeedant and nematicidal effects of **4**. Phenolic acids related to **5** and **6** are known for their insecticidal activities [41] and play an important role in plant resistance against insect pests [42]. For example, previous reports showed that phenylacetic compounds, isolated from *Streptomyces gramineus*, have insecticidal activity against *Thrips palmi*, also known as melon thrips, a sap-sucking phytophagous insect [43].

The phytotoxic effects of **3** and **4** are consistent with those of the analogous compounds of alkylitaconic acid derivatives, which promoted radicle growth at low doses while inhibiting the seedlings’ growth at high doses, particularly in dicotyledonous species [36,44]. Additionally, Mondal et al. [45] reported that **4** stimulated germination and seedling growth in cauliflower at low ppm concentrations. This behavior could be explained by the hormetic effects of toxic agents showing a biphasic response, promoting early seedling development (and potentially later plant growth) at low concentrations while inhibiting growth at high concentrations [46,47]. This work has shown for the first time the potent phytotoxic effects of (+)-piliformic acid (**3**) and hexylaconitic A anhydride (**4**) on mono- and dicotyledonous plants (*L. perenne* and *L. sativa*) and stimulating effect on the growth of *L. sativa* root at low doses.

Overall, compound **4** (hexylaconitic acid A anhydride)**,** the major metabolite of the extract, was active on all the targets (except *S. littoralis*) with efficient doses above the positive control in all the cases (between 1 and 22 times the activity of thymol) and lower than the phytotoxic concentration (0.2 mg/mL). Therefore, improved fermentation methods aimed to increase the concentration of **4** are needed prior to the upscaled fermentation processes needed to produce this new biocontrol agent.

## 3. Materials and Methods

### 3.1. General Experimental Procedures

Optical rotations were measured on an MCP 150-Anton Paar polarimeter (Anton Paar, Seelze-Letter, Germany) at a temperature of 25 °C. The IR spectra were recorded with a Cary 630 FTIR spectrophotometer (Agilent, Santa Clara, CA, USA). The ECD spectra were recorded on a JASCO J-1500 CD spectrometer (JASCO, Tokyo, Japan). The ^1^H, ^13^C, and 2D (COSY, gHSQC, HMBC, and NOESY) NMR spectra were recorded in CDCl_3_ or CD_3_OD solution (Aldrich, St. Louis, MO, USA) on a Bruker Avance II-500 spectrometer equipped with a 5 mm TCI inverse detection cryo-probe (Bruker Biospin, Falländen, Switzerland). Chemical shifts are given in ppm (δ), referenced to solvent signal (CDCl_3_, δ_H_ 7.26 and δ_C_ 77.0; CD_3_OD_3_, δ_H_ 3.31 and δ_C_ 49.0). The HRESIMS mass spectra were obtained using a Waters LCT Premier XE mass spectrometer (Manchester, UK). Sephadex LH-20 (Sigma-Aldrich, St. Louis, MO, USA) and silica gel 60 (40 ± 63 μm, Merck, Darmstadt, Germany) were used for column chromatography. TLC was performed with precoated silica gel 60 F254 plates (Merck, Darmstadt, Germany). Flash chromatography separations were carried out on a Biotage Isolera Prime (BIOTAGE, Uppsala, Sweeden) apparatus equipped with a UV detector (200–400 nm) using a prepacked RediSep flash column of silica gel (12 × 3 cm, 35 g Si; Teledyne Isco, Lincoln, NEB, USA). Semipreparative HPLC was performed on a Beckman System Gold 125P equipped with a diode-array detector Beckman Coulter 168 (Brea, CA, USA) using a semi-preparative Beckman Ultrasphere ODS (10.0 × 250 mm, 5 μm) column.

### 3.2. Lichen Material and Isolation of the Endolichenic Fungus HYP6

The fungal strain HYP6 was isolated from the lichen *Hypogymnia tubulosa* collected in Tenerife Island (Las Lagunetas, 28°24′47.2″ N, 16°24′11.2″ W) from the bark of endemism *Pinus canariensis*, the dominant tree of the Canarian pine forest ecosystem. The samples were placed into sterile polybags and transported under refrigeration in a box container until isolation processing within 24 h of collection. The collected lichen thalli were initially washed with distilled water to remove excess dirt. For surface sterilization, thalli were cut into manageable portions followed by successively dipping in a 70% ethanol solution for 1 min, 1% sodium hypochlorite for 10 min, and finally washing with 70% ethanol for 1 min. The surface-sterilized thalli were dried on sterilized paper and cut into 1cm pieces. Then, lichen explants were inoculated in Potato Dextrose Agar (PDA) and Malt Extract Agar (MEA) media in Petri dishes containing 50 mg/L of the antibiotic chloramphenicol to avoid bacterial contamination. The plates were incubated at 24 °C for 2–3 weeks in darkness with daily observation of the emerging fungal colonies. The new fungal mycelia that grew from the lichen explants were transferred to fresh plates containing the respective medium and various subcultures were performed until pure cultures were obtained.

### 3.3. Molecular Characterization of HYP6 Strain

For the extraction of the DNA of the fungal strain, the DNeasy Plant mini kit (Qiagen GmbH, Hilden, Germany, Cat. No 69104) was used. The ITS rDNA region was PCR-amplified using the oligonucleotide primers ITS1 (5′-TCCGTAGGTGAACCTGCGG-3′) and ITS4 (5′-TCCTCCGCTTA TTGATATGC-3′) with the following steps: Genomic DNA (100–200 ng) was amplified on a PTC-200 Thermal Cycler (MJ Research, San Diego, CA, USA) in a 25 µL final volume with the AmpONE Taq DNA polymerase PCR kit (GeneAll, Seoul, Korea) for 35 cycles (95 °C, 1 min; 50 °C, 20 s; 72 °C, 1.5 min) after an initial denaturation (95 °C, 2 min) and followed by a final extension (72 °C, 7 min). The amplicons were checked by agarose gel (1%) electrophoresis, purified using the EXO-SAP-IT kit (Affimetrix-USB; Thermo Fisher Scientific, Waltham, MA, USA), and sequenced on an AB 3500 Genetic Analyzer (Thermo Fisher Scientific, Waltham, MA, USA) at the University of La Laguna (La Laguna, Spain) genomic service. The consensus sequence of the rDNA ITS region was generated using the aligner software Bioedit (version 5.09; Tom Hall, Department of Microbiology, North Carolina State University, Raleigh, NC, USA) and compared with those published in the NCBI (https://www.ncbi.nlm.nih.gov/) database by using the online BLAST program (https://www.ncbi.nlm.nih.gov/BLAST, accessed on 29 May 2024). The related sequences with high percent identity were aligned using the MEGA11.0 program (Mega Limited, Auckland, New Zealand, Tamura K. 2021) [48] and a phylogenetic tree was constructed using the maximum likelihood method with a Tamure-3 parameter (T92+G) model and a bootstrap test with 5000 runs. The display and annotation of the phylogenetic tree were performed using the online tool The Interactive Tree Of Life (https://itol.embl.de, accessed on 31 May 2024).

### 3.4. Cultivation of HYP6

The endolichenic strain HYP6 was cultivated on a PDA solid medium for 10 days at 25 °C. Sterile water (10 mL) was added to each Petri dish and the surface of the mycelium was gently scraped with a spatula to obtain a suspension of the mycelium. This suspension was cultivated in 250 mL Erlenmeyer flasks containing 50 mL of MEB medium (17 g/L of malt extract, 3 g/L of peptone, pH adjusted to 5.4 ± 0.2) for 5 days at 25 °C on a rotary shaker (120 rpm) to prepare the seed culture. Ten Erlenmeyer flasks (500 mL) with 200 mL of fresh MEB medium each were inoculated with 10 mL of seed culture and cultured at 25 °C under continuous agitation (120 rpm) for 14 days.

### 3.5. Extraction, Isolation and Characterization of Compounds

The fermentation broth was filtered under reduced pressure through a Buchner funnel using cheesecloth (25 µm pore diameter) to separate the mycelium. The resulting filtrate was subjected to liquid/liquid extraction (three times) with ethyl acetate (EtOAc), dried over anhydrous Na_2_SO_4_, and the organic phase was concentrated under reduced pressure to yield the crude extract (817.5 mg).

The crude EtOAc extract was fractionated by the column chromatography of silica gel eluting with a gradient of increasing polarity of n-hexane/EtOAc and then CH_2_Cl_2_/MeOH. The fractions obtained were analyzed by TLC and those with high similarity were combined to afford five main fractions. Fraction 2 was separated by medium-pressure liquid chromatography using a Biotage Isolera Prime equipment with a 35 g Si pre-packed flash cartridge column, eluted with n-hexane/EtOAc mixtures of increasing polarity (70:30–25:75) at 10 mL/min to afford **3** (33.7 mg) and **4** (13.5 mg). Fraction 3 was purified by the Sephadex LH-20 column and eluted with a mixture of CH_2_Cl_2_:MeOH (1:1) to give **5** (11.3 mg) and **6** (8.4mg). Fraction 4 was separated by the Sephadex-LH-20 column using an isocratic mixture of n-hexane:CH_2_Cl_2_:MeOH (1:1:1) to yield four subfractions (SFrs. 4.1–4.4). SFr.4.3 was further purified by reverse-phase semi-preparative HPLC (Ultrasphere ODS, 10.0 × 250 mm, 5 μm) using a gradient of H_2_O/MeOH (80:20–30:70) at a flow rate of 3 mL/min to afford **2** (18.4 mg, t_R_ = 42.6 min). Similarly, the reverse-phase semi-preparative HPLC of subfraction SFr.4.4 eluted with H_2_O:MeOH (60:40) gave **1** (4.6 mg, t_R_ = 32.0 min).

#### 3.5.1. (+)-9-Hydroxypiliformic Acid (**1**)

Colourless oil; $[\alpha {]}_{D}^{25}$ +16 (c 0.5, CHCl_3_); IR ν_max_: 3355, 2940, 2834, 1689, 1637, 1215, 1020 cm^−1^; ^1^H NMR data (CD_3_OD, 500 MHz,) see Table 1 and Figure S1; ^13^C NMR data (CD_3_OD, 125 MHz) see Table 1 and Figure S2; HRMS (ESI^+^) *m/z* 253.1059 [M + Na]^+^ (calcd. C_11_H_18_O_5_Na, 253.1052).

#### 3.5.2. (+)-8-Hydroxypiliformic Acid (**2**)

Colourless oil; $[\alpha {]}_{D}^{25}$ +30 (c 0.5, CHCl_3_); IR ν_max_: 3324, 2945, 2849, 1684, 1636, 1226, 1015 cm^−1^; ^1^H NMR data (CD_3_OD, 500 MHz) see Table 1 and Figure S9; ^13^C NMR data (CD_3_OD, 125 MHz) see Table 1 and Figure S10; HRMS (ESI^+^) *m/z* 253.1058 [M + Na]^+^ (calcd. C_11_H_18_O_5_Na, 253.1052).

### 3.6. Antifungal Bioassay

To evaluate the antifungal activity, three different phytopathogenic fungi were used as follows: *Fusarium oxysporum*, *Alternaria alternata*, and *Botrytis cinerea*. These strains came from the fungal collection at Instituto de Productos Naturales y Agrobiologia-CSIC (Tenerife, Spain), and the colonies were maintained on PDA medium plates in darkness at 25 °C.

Based on the protocol described before [49], the in vitro mycelial growth inhibition assay was conducted in 12-well plates (Falcon) using a modified PDA-dilution method supplemented with the addition of 0.05 mg/mL of methyltetrazolium salts (MTT). The extract and pure compounds dissolved in ethanol (EtOH) were tested at different concentrations (extract at 1 mg/mL and compounds at 0.5, 0.25, 0.1, and 0.05 mg/mL) and were incorporated into the culture medium before the plates were poured. EtOH was used as a negative control and all the treatments were replicated four times. Fungal colonies were digitalized and measured using ImageJ (http://imagej.nih.gov/ij/, accessed on 18 March 2024) [50] after the incubation of the plates at 25 °C in darkness for 48 h. The mycelial growth inhibition (% MGI) was calculated as follows: % MGI = (C − T/C) × 100, where C is the diameter of the control colonies and T is the diameter of the test colonies. Data were analyzed with the STATGRAPHICS statistical analysis software (Centurion XVI, version 16.1.03). A nonparametric Kruskal–Wallis ANOVA analysis of variance was performed on mycelial growth data, and the means were compared by the Mann–Whitney U test at *p* < 0.05. The EC_50_ values (effective dose to obtain 50% of inhibition) were determined by regression analysis (% MGI on Log-dose). Thymol (Sigma-Aldrich) was used as a positive control with EC_50_ values of 2.0, 1.3, and 2.6 µg/mL against *F. oxysporum*, *A. alternata*, and *B. cinerea*, respectively.

### 3.7. Antifeedant Bioassay

*Spodoptera littoralis* colonies were reared on an artificial diet [51], while *Myzus persicae* and *Rhopalosiphum padi* colonies were maintained on bell pepper (*Capsicum annuum*) and barley *(Hordeum vulgare*) plants, respectively. The plants were grown from seeds in pots with commercial substrate and regularly infected for aphid feeding (bell pepper plants were infected at a 4-leaf stage and barley plants when they reached approximately a length of 10 cm). Both the insect colonies and their host plants were maintained in a growth chamber at 22 ± 1 °C, >70% relative humidity with a 16:8 h light photoperiod.

Bioassays were conducted with 1.0 cm^2^ leaf disks/fragments of *C. annuum* (*M. persicae*, *S. littoralis*) or *H. vulgare* (*R. padi*) as described previously [52]. The tests (10 μL of the solution in EtOH) were applied at initial doses of 10 or 5 mg/mL (extract or compound) to the upper surface of the leaf fragments. Two sixth-instar larvae (>24 h after molting) of *S. littoralis* were placed in 6 Petri dishes (9 cm in diameter) with 2 leaf disks (treatment disk with the test solution and control disk with solvent) and allowed to feed at room temperature until 75% larval consumption of the paired control or treatment disks. The leaf disk surface consumption was measured using ImageJ (http://imagej.nih.gov/ij/, accessed on 18 March 2024) [49]. In the case of aphids, twenty (2 × 2 cm) ventilated plastic boxes containing 10 apterous aphid adults (24–48 h old) were used. The aphids were allowed to feed in a growth chamber under the described environmental conditions for 24 h. Settling was quantified by counting the number of aphids settled on each leaf fragment. All the experiments were repeated twice (SE < 10%).

The feeding or settling inhibition (%FI or %SI) was calculated as [1 − (T/C) × 100], where T and C represent the treated and control leaf fragments, respectively. The effects (%SI/%FI) were analyzed by the nonparametric Wilcoxon Signed-Rank Test. Compounds with an effect ≥70% were tested in dose–response experiments (3–5 serial dilutions) to calculate their EC_50_ (the effective dose causing a 50% settling/feeding reduction) with linear regression models (%FI/SI on Log-dose). The positive control was thymol (Sigma Aldrich) with EC_50_ values of 7.6 and 18.6 μg/cm^2^ for *M. persicae* and *R. padi.*

### 3.8. Nematicidal Bioassay

The *Meloidogyne javanica* population was maintained on tomato plants (*Solanum lycopersicum* var. Marmande) cultivated in pot cultures and kept in environmentally controlled growth chambers (at 25 ± 1 °C, >70% relative humidity). Egg masses of *M. javanica* were handpicked from the infected tomato roots two months after seedling inoculation. Second-stage juveniles (J2) were obtained by incubating egg masses in a water suspension at 25 °C for 24 h. The tests were carried out in 96-well plates (BD Falcon, San Jose, CA, USA) and the extract and compounds were dissolved in distilled water containing 5% of a DMSO-Tween solution (0.2% Tween 20 in DMSO) according to Andres et al. [53]. The initial concentrations tested were 1 and 0.5 mg mL for the extract and pure compound, respectively, and four replicates were used for each test. Water containing 5% of a DMSO-Tween solution (0.2% Tween 20 in DMSO) was used for the negative control. The mortality rates after 72 h of incubation are presented as a percentage of dead J2 corrected according to Scheider-Orelli’s formula. The result values were analyzed by ANOVA, and the means were compared by LSD at *p* < 0.05. Serial dilutions were used to calculate the effective lethal doses (LD_50_) of the active compound by Probit Analysis (STATGRAPHICS Centurion XVI, version 16.1.03). Thymol (Sigma Aldrich) was used as a positive control with an LD_50_ value of 0.14 mg/mL.

### 3.9. Phytotoxic Bioassay

The phytotoxic test was conducted with *Lolium perenne* and *Lactuca sativa* seeds placed in 12-well microplates (40 seeds for the test), as described in [54]. The extract or compounds dissolved in EtOH (negative control) were tested at concentrations of 0.4 or 0.2 mg/mL (final concentration in the well) and diluted serially if needed. Juglone (Sigma) was used as a positive control (0.1 mg/mL), resulting in 100% germination inhibition. Briefly, the test solution (20 µL) and 300 µL of H_2_O were added to a 2.5 cm diameter filter paper into each well plate. The seeds (10/5 of *L. sativa*/*L. perenne* soaked in distilled water for 8 h) were placed in every well and the parafilm-sealed plates were incubated in a plant growth chamber (25 °C, 70% RH, 16:8 L:D). Germination was monitored for 7 days and leaf length (for *L. perenne*) and root length (for both species) were measured at the end of the experiment on 25 randomly selected digitalized seedlings with the ImageJ application (http://rsb.info.nih.gov/ij/, acceded on 20 May 2024) [50]. A nonparametric Kruskal–Wallis ANOVA analysis of variance was performed using the STATGRAPHICS statistical analysis software (Centurion XVI, version 16.1.03) on root/leaf length data, and the means were compared by the Mann–Whitney U test at *p* < 0.05.

## 4. Conclusions

In this study, the endolichenic fungus HYP6, isolated from the epiphytic lichen *Hypogymnia tubulosa*, was identified as *Xylaria* sp. belonging to the *X. arbuscula* complex. The EtOAc extract from the liquid culture was chromatographed to afford (+)-piliformic acid (**3**) as the major metabolite and two unreported hydroxyl derivatives of **3**, (+)-9-hydroxypiliformic acid (**1**) and (+)-8-hydroxypiliformic acid (**2**), together with the previously reported compounds, hexylaconitic acid A anhydride (**4**) and the hydroxyphenylacetic derivatives (**5** and **6**). The extract showed significant aphid antifeedant, antifungal, and phytotoxic effects. The aphid antifeedant activity of the extract against *M. persicae* can be explained by the active compounds **4**–**6**, whereas its moderate activity against *R. padi* is probably due to its content of antifeedant compound **4**. Compound **4** also showed potent effects against *B. cinerea* and the nematode *M. javanica*. The phytotoxicity observed can be attributed to compounds **3**–**5**, affecting negatively the growth of *L. perenne* and *L. sativa*. In addition, **3** and **4** showed stimulating effects on the growth of *L. sativa* root at low doses. The endolichenic fungus *Xylara* sp. represents an important source for the biotechnological production of biopesticide compounds for plant disease control. Further optimization of the fermentation process is needed to improve the production parameters (yield, fermentation time) of these active metabolites.

## Figures and Tables

**Table 1:** ^1^H NMR (500 MHz) and ^13^C NMR (125 MHz) spectra for **1** and **2** in CD_3_OD.

Position	1		2	
	δ_H_, mult (*J* in Hz)	δ_C_, Type	δ_H_, Mult (*J* in Hz)	δ_C_, Type
**1**	-	178.0, C	-	177.8, C
**2**	3.63, q (7.1)	39.0, CH	3.64, qd (7.1, 1.9)	38.7, CH
**3**	-	134.5, C		134.3, C
**4**	6.82, t (7.6)	144.7, CH	6.85, t (7.6)	145.0, CH
**5**	2.26, m	29.4, CH_2_	2.26, m	29.4, (29.4) ^a^, CH_2_
**6**	1.51, m	29.6, CH_2_	1.61, m 1.52, m	26.0, (26.0) ^a^, CH_2_
**7**	1.43, m	26.7, CH_2_	1.50, m	39.6, (39.7) ^a^, CH_2_
**8**	1.56, m	33.4, CH_2_	3.74, sext (6.1)	68.2,(68.3) ^a^, CH
**9**	3.55, t (6.6)	62.8, CH_2_	1.16, d (6.1)	23.5, CH_3_
**10**	-	170.3, C	-	169.9, C
**11**	1.31, d (7.1)	16.4, CH_3_	1.31, d (7.1)	16.3, CH_3_

^a^ data in () for stereoisomer of hydroxyl group in n-alkyl chain.

**Table 2:** Biocidal effects of the extract and compounds (**2**–**6**) against insect pests (*Spodoptera littoralis*, *Myzus persicae*, and *Rhoplalosiphum padi*), the nematode *Meloidogyne javanica*, and phytopathogenic fungi (*Alternaria alternata*, *Botrytis cinerea*, and *Fusarium oxysporum*).

Target	Effect	Extract/Compound	Activity (%)	EC_50_^c^/LD_50_^d^
***S. littoralis***	%FI ^a^	**Extract**	42.9 ± 11.4	
		**2**	33.0 ± 14.9	
		**3**	18.0 ± 8.4	
		**4**	37.8 ± 18.7	
		**5**	9.1 ± 6.3	
		**6**	17.7 ± 11.7	
***M. persicae***	%SI ^a^	**Extract**	81.5 ± 6.8 *	
		**2**	25.5 ± 6.3	
		**3**	50.4 ± 8.9	
		**4**	100.0 ± 0.0 *	1.6 (0.5–4.9)
		**5**	98.9 ± 0.6 *	4.5 (2.0–10.0)
		**6**	91.9 ± 2.3 *	15.5 (12.0–20.0)
***R. padi***	%SI ^a^	**Extract**	61.5 ± 6.0 *	
		**2**	20.6 ± 6.4	
		**3**	30.3 ± 8.8	
		**4**	91.0 ± 3.8 *	8.9 (4.6–7.5)
		**5**	61.2 ± 9.2 *	
		**6**	43.0 ± 7.9	
***M. javanica***	%Mortality ^b^	**Extract**	2.0 ± 0.7	
		**2**	2.2 ± 0.7	
		**3**	28.4 ± 6.6 *	
		**4**	100.0 ± 0.0 *	0.10 (0.10–0.11)
		**5**	26.4 ± 5.8 *	
		**6**	29.5 ± 5.6 *	
***A. alternata***	% MGI ^b^	**Extract**	58.5 ± 2.4 *	
		**2**	27.3 ± 3.7	
		**3**	47.3 ± 5.1	
		**4**	63.7 ± 1.4 *	0.24 (0.20–0.28)
		**5**	32.2 ± 5.6	
		**6**	42.5 ± 6.0	
***B. cinerea***	% MGI ^b^	**Extract**	75.9 ± 9.7 *	
		**2**	19.7 ± 6.2	
		**3**	62.1 ± 7.6 *	0.36 (0.25–0.51)
		**4**	84.3 ± 4.5 *	0.12 (0.09–0.15)
		**5**	0.5 ± 8.8	
		**6**	5.8 ± 6.2	
***F. oxysporum***	% MGI ^b^	**Extract**	61.9 ± 6.5 *	
		**2**	18.7 ± 3.1	
		**3**	42.3 ± 3.6	
		**4**	52.3 ± 2.6 *	
		**5**	32.6 ± 9.1	
		**6**	35.7 ± 3.3	

^a^ feeding/settling inhibition (%FI/%SI) of extract (100 μg/cm^2^) and compounds (50.0 μg/cm^2^). ^b^ J2 Mortality (%) and mycelial growth inhibition (% MGI) of extract (1.0 mg/mL) and compounds (0.5 mg/mL). ^c^ effective EC_50_ doses (feeding/settling in μg/cm^2^, % MGI in mg/mL) and 95% confidence limits (lower-upper). ^d^ effective lethal dose LD_50_, (mg/mL) and 95% confidence limits (lower-upper).* *p* < 0.05: Wilcoxon Signed-Rank Test (antifeedant tests), Mann–Whitney U test (fungicidal tests), and Student *T*-test (nematicidal test).

## Data Availability

Data are contained within the article and Supplementary Materials.

## Supplementary Materials

The following supporting information can be downloaded at https://www.mdpi.com/article/10.3390/molecules30030470/s1, Data sequence of *Xylaria* sp.; Figures S1–S8: ^1^H-NMR, ^13^C-NMR, COSY, gHSQC, HMBC, NOESY, HREIMS, and IR spectra for compound **1;** Figures S9–S16: ^1^H-NMR, ^13^C-NMR, COSY, gHSQC, HMBC, NOESY, HREIMS, and IR spectra for compound **2**; Figures S17–S22: ^1^H-NMR, ^13^C-NMR, COSY, gHSQC, HMBC, and NOESY spectra for compound **4**; Figure S23: ECD spectra of compounds **1**–**2**. Table S1: ^1^H and ^13^C-NMR spectroscopic data for compound **4**; Figure S24: Phytotoxic effects of compounds **3** and **4** on *L. perenne* leaf and root growth and *L. sativa* root growth in lower doses.
