# Supplementary material for: Biopesticide Compounds from an Endolichenic Fungus Xylaria sp. Isolated from the Lichen Hypogymnia tubulosa
Source: Molecules. 2025 Jan 22;30(3):470. doi: 10.3390/molecules30030470 (PMC11820368; doi:10.3390/molecules30030470)
Supplement: Supplementary file 1 [file molecules-30-00470-s001.zip › molecules-3318145-supplementary.pdf]

Biopesticide Compounds from an Endolichenic Fungus *Xylaria sp.* Isolated from the Lichen *Hypogymia tubulosa*

Fotios A. Lyssaios [1], Azucena González-Coloma [2,*], María Fe Andrés [2], Carmen E. Díaz [1,*]

[1] *Instituto de Productos Naturales y Agrobiología, CSIC, Avda. Astrofísico F. Sánchez 3, 38206 La Laguna, Tenerife, Spain*

[2] *Instituto de Ciencias Agrarias, CSIC, Serrano 115-dpdo, 28006 Madrid, Spain*

*Corresponding authors:

E-mail address: celisa@ipna.csic.es (C.E. Díaz)

E-mail address: azu@ica.csic.es (A. González-Coloma)

List of contents:

Sequence data of HYP6 (*Xylaria sp.*).

Figure S1. ^{1}H-NMR spectrum of compound **1** (CD_3OD, 500 MHz)

Figure S2. ^{13}C-NMR spectrum of compound **1** (CD_3OD, 125 MHz)

Figure S3. ^{1}H-^{1}H COSY spectrum of compound **1** (CD_3OD, 500 MHz)

Figure S4. gHSQC spectrum of compound **1** (CD_3OD, 500 MHz)

Figure S5. HMBC spectrum of compound **1** (CD_3OD, 500 MHz)

Figure S6. NOESY spectrum of compound **1** (CD_3OD, 500 MHz)

Figure S7. HRESIMS of compound **1**

Figure S8. IR spectrum of compound **1**

Figure S9. ^{1}H-NMR spectrum of compound **2** (CD_3OD, 500 MHz)

Figure S10. ^{13}C-NMR spectrum of compound **2** (CD_3OD, 125 MHz)

Figure S11. ^{1}H-^{1}H COSY spectrum of compound **2** (CD_3OD, 500 MHz)

Figure S12. gHSQC spectrum of compound **2** (CD_3OD, 500 MHz)

Figure S13. HMBC spectrum of compound **2** (CD_3OD, 500 MHz)

Figure S14. NOESY spectrum of compound **2** (CD_3OD, 500 MHz)

Figure S15. HRESIMS of compound **2**

Figure S16. IR spectrum of compound **2**

Figure S17. ^{1}H-NMR spectrum of compound **4** ($CDCl_3$, 500 MHz)

Figure S18. ^{13}C-NMR spectrum of compound **4** ($CDCl_3$, 125 MHz)

Figure S19. ^{1}H-^{1}H COSY spectrum of compound **4** ($CDCl_3$, 500 MHz)

Figure S20. gHSQC spectrum of compound **4** ($CDCl_3$, 500 MHz)

Figure S21. HMBC spectrum of compound **4** ($CDCl_3$, 500 MHz)

Figure S22. NOESY spectrum of compound **4** ($CDCl_3$, 500 MHz)

Figure S23. ECD spectra of compounds **1-2**

Table S1. ^{1}H (500MHz) and ^{13}C (125 MHz) spectroscopic data for compound **4** in $CDCl_3$

Figure S24. Phytotoxic effects of compounds **3** and **4** on *L. perenne* leaf and root growth and *L. sativa* root growth in lower doses

Sequence data of fungal strain HYP6 *Xylaria sp.*

TTCCGTAGGTGAACCTGCGGAGGGATCATTAAAGAGTTTACACAACTCCTAAA
CCCATGTGAACTTMCCTTTTGTTGCCTCGGCAGGTCGCGTTTACCCTGTGAGGG
CCTACCCTGTAGGCTCTTACCTGGTGGACGTGGGCTCCCCTGCCGGCGGCCCGT
TAAATTCTGTTTATTATATTATTTCTGAATCTATAACTAAATAAGTTAAAACTTT
CAACAACGGATCTCTTGGTTCTGGCATCGATGAAGAACGCAGCGAAATGCGAT
AAGTAATGTGAATTGCAGAATTCAGTGAATCATCGAATCTTTGAACGCACATTG
CGCCCATTAGTATTCTAGTGGGCATGCCTGTTCGAGCGTCATTTCAACCCTTAA
GCCTCTGTTGCTTAGTGTTGGGAGCCTACAGCCCTCTGTAGCTCCCCAAAGTTA
GTGGCGGAGTCGGTTCACACTCTAGACGTAGTAAATTTTTATCTCGCCTWTAGA
TGAGCCGGCGCCTTGCCGTAAAACCCCCTAATTTTTCACAAGGKTGACCTCGGA
TCAGGKAGGAATACCCGCTGAACTTAAGCATATCAATA

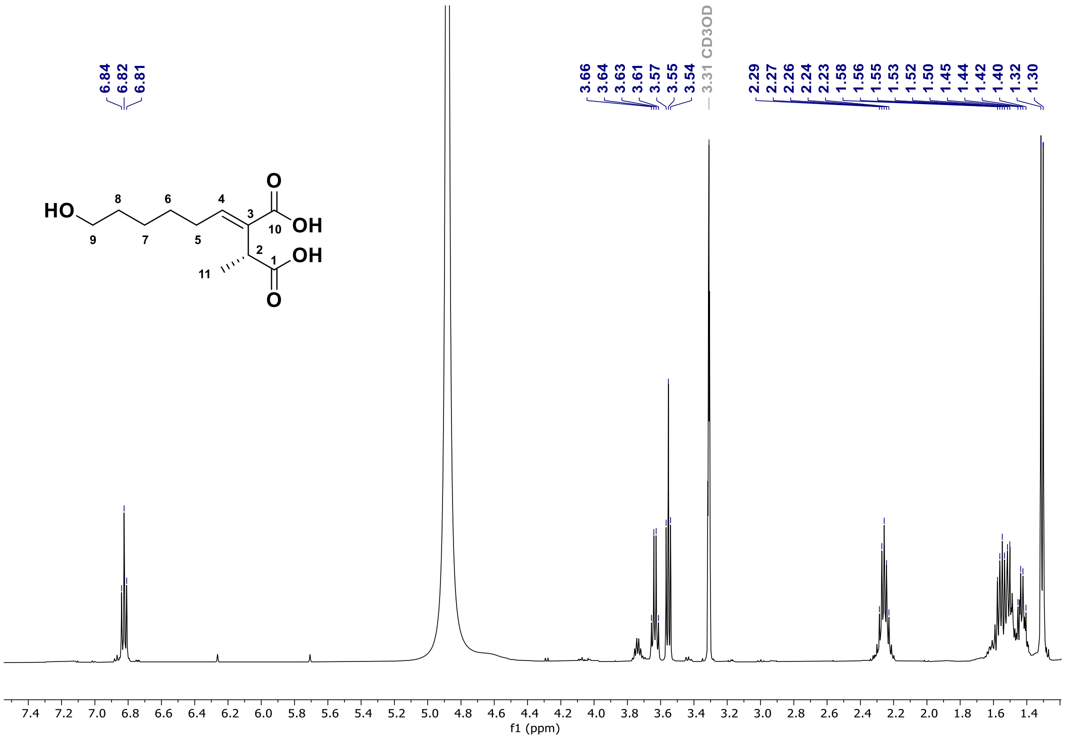

Figure S1. ^{1}H-NMR spectrum of compound **1** (CD_3OD, 500 MHz)

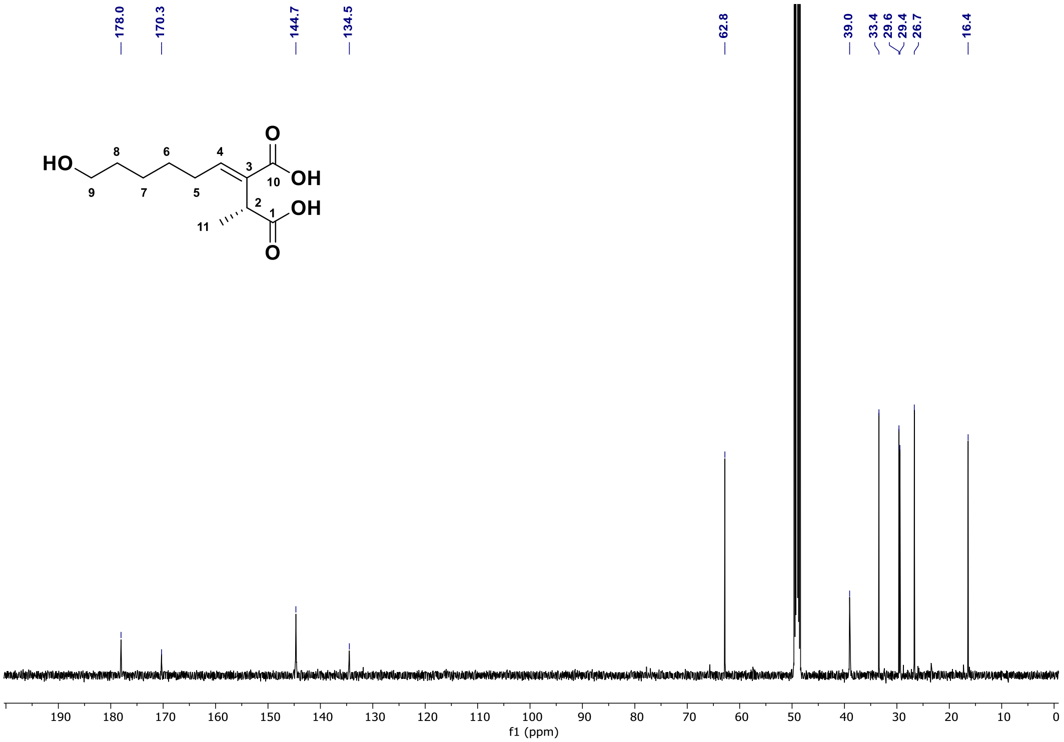

Figure S2. ^{13}C-NMR spectrum of compound **1** (CD_3OD, 125 MHz)

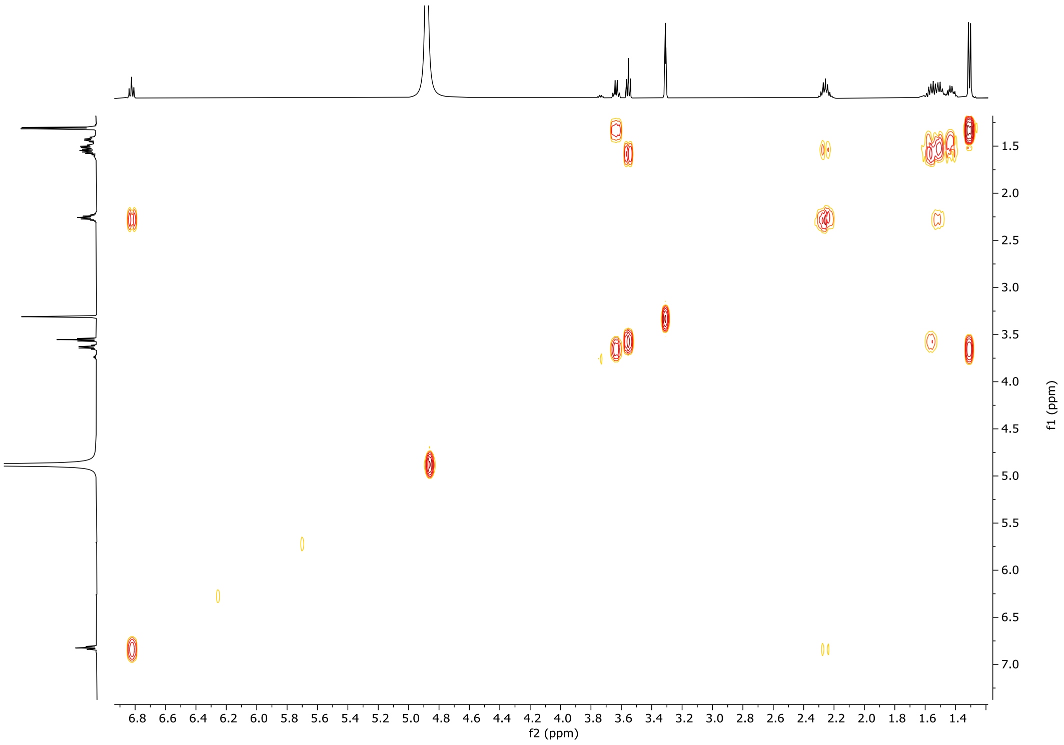

Figure S3. ^{1}H-^{1}H COSY spectrum of compound **1** (CD_3OD, 500 MHz)

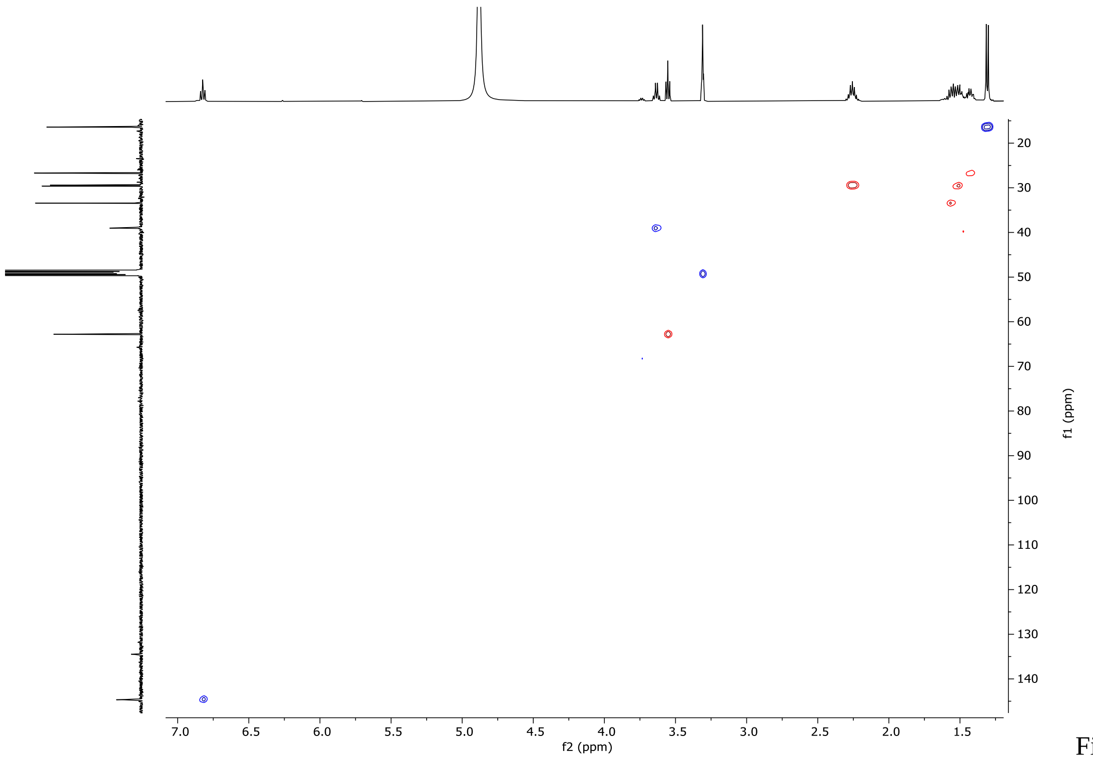

Figure
S4. gHSQC spectrum of compound 1 (CD_3OD, 500 MHz)

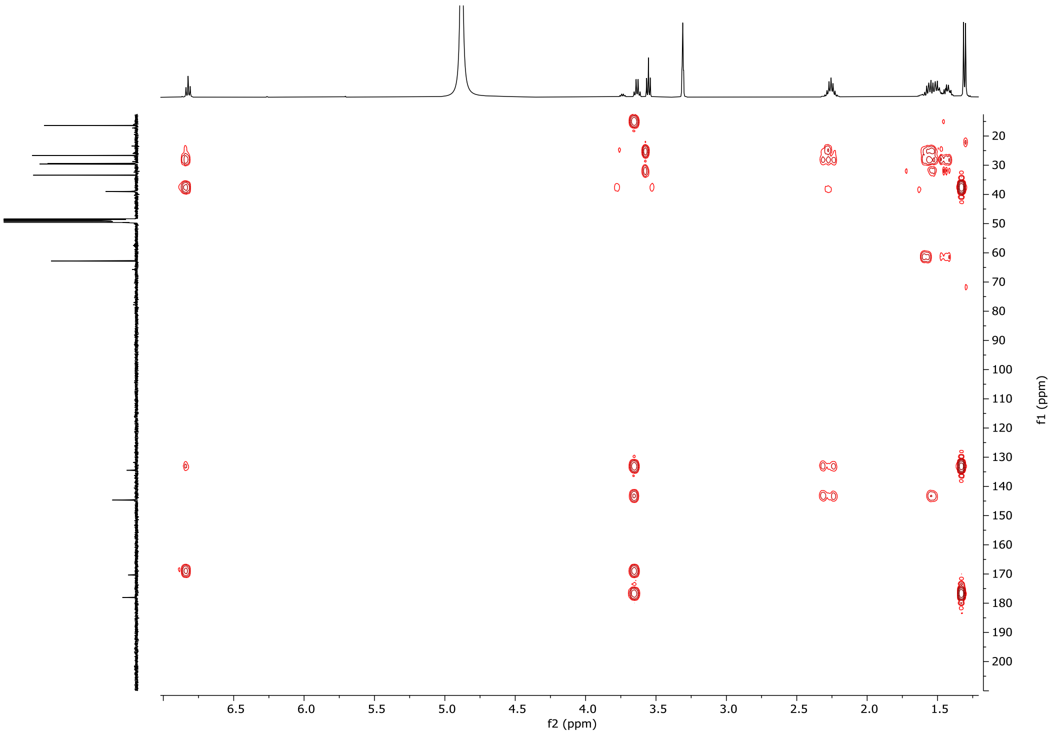

Figure S5. HMBC spectrum of compound **1** (CD_3OD, 500 MHz)

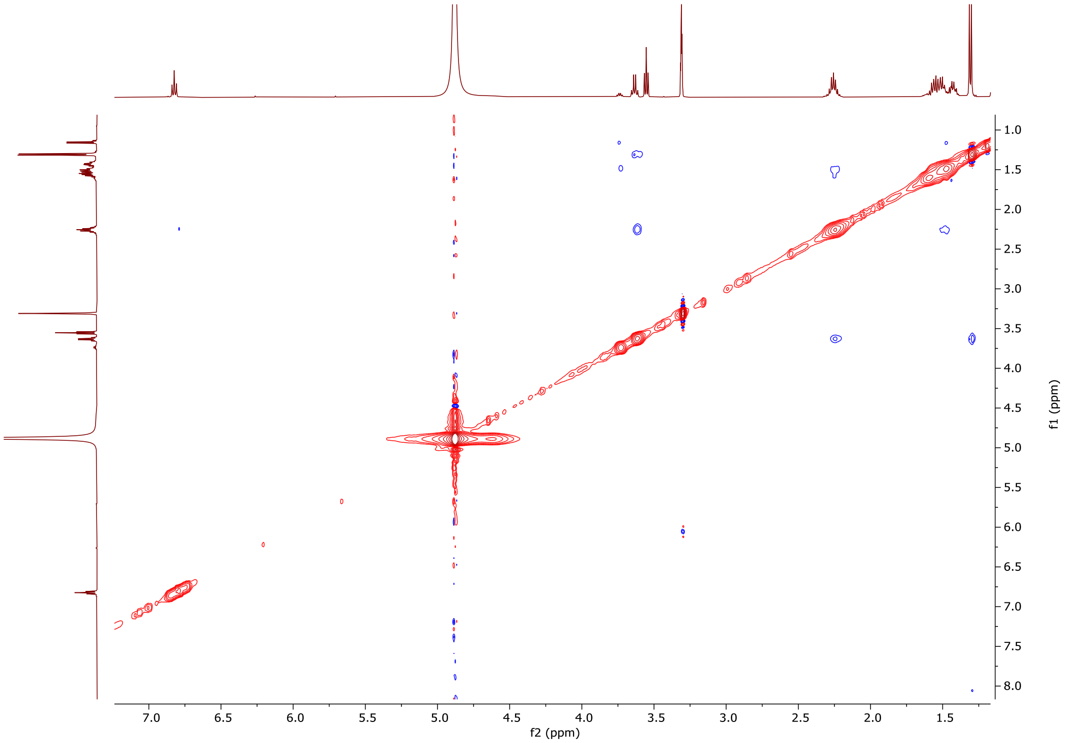

Figure S6. NOESY spectrum of compound **1** (CD_3OD, 500 MHz)

Elemental Composition Report **Page 1**

Tolerance = 5.0 PPM / DBE: min = -1.5, max = 100.0
Element prediction: Off
Number of isotope peaks used for i-FIT = 3

Monoisotopic Mass, Even Electron Ions
892 formula(e) evaluated with 5 results within limits (up to 50 best isotopic matches for each mass)
Elements Used:
C: 0-58 H: 0-70 N: 0-8 O: 0-11 Na: 0-1 S: 0-1
Carmen E
ESI (22-070) Carmen E (HYP6C7I 48-50) 45 (1.951) 1: TOF MS ES+ 2.15e+002

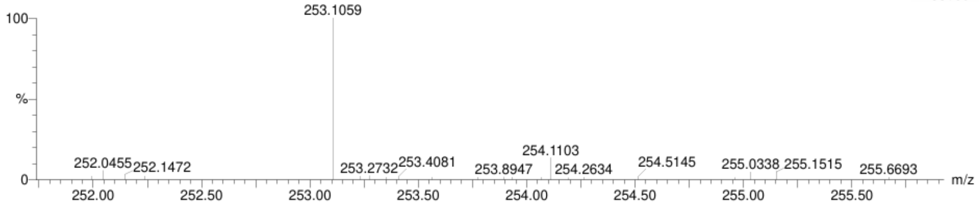

Minimum:	30.00				-1.5			
Maximum:	100.00		5.0	5.0	100.0			
Mass	RA	Calc. Mass	mDa	PPM	DBE	i-FIT	i-FIT (Norm)	Formula
253.1059	100.00	253.1052	0.7	2.8	2.5	54.2	1.1	C11 H18 O5 Na
		253.1065	-0.6	-2.4	7.5	54.6	1.4	C12 H14 N4 O Na
		253.1049	1.0	4.0	6.5	54.7	1.6	C9 H13 N6 O3

Figure S7. HRESIMS of compound **1**

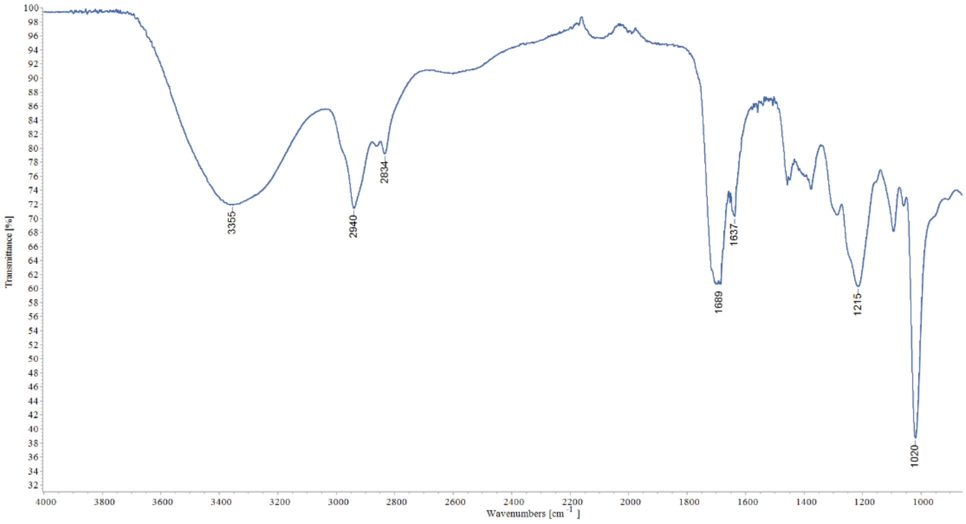

Figure S8. IR spectrum of compound **1**

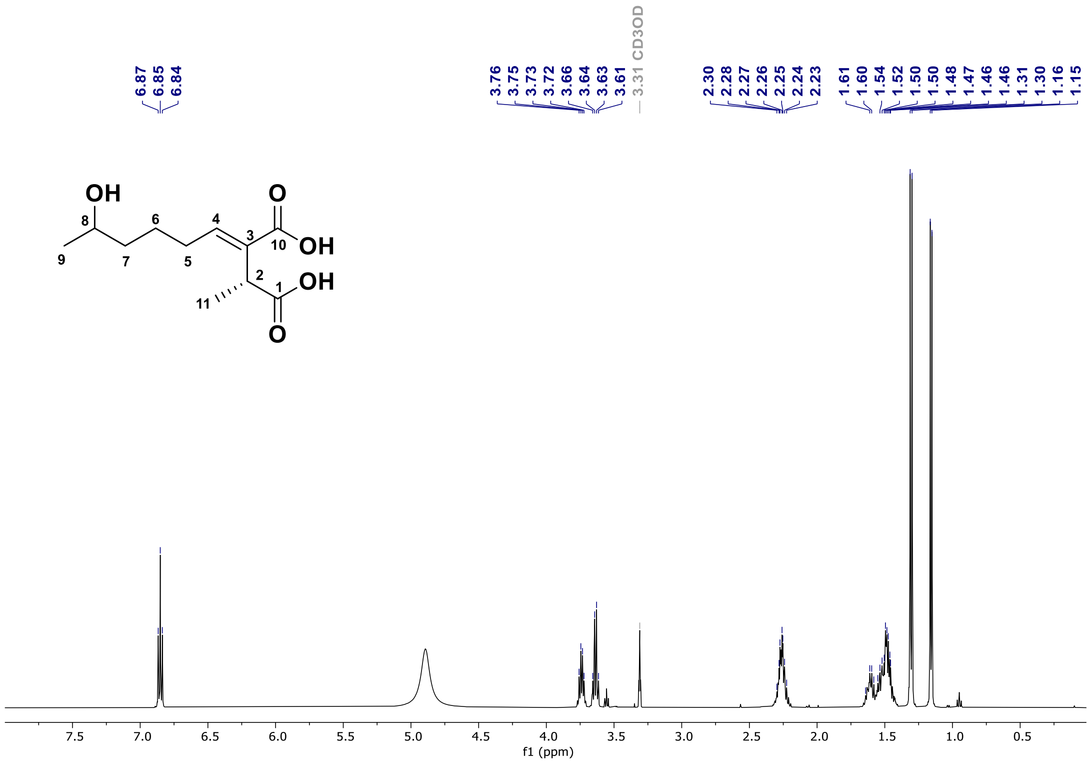

Figure S9. ^{1}H-NMR spectrum of compound **2** (CD_3OD, 500 MHz)

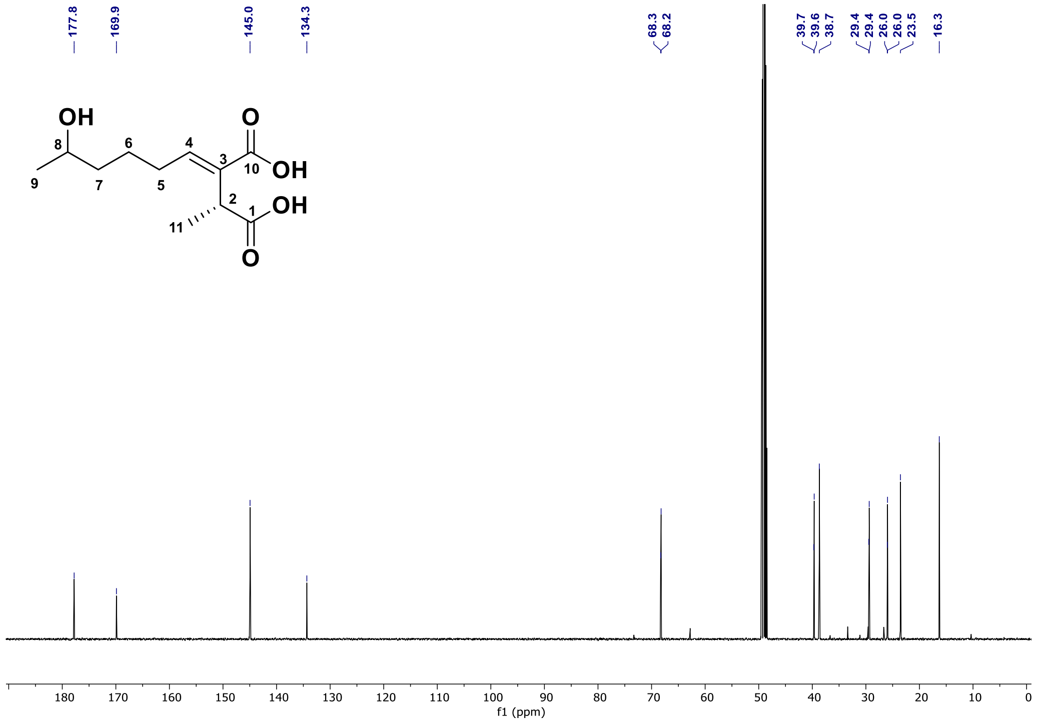

Figure S10. ^{13}C-NMR spectrum of compound **2** (CD_3OD, 125 MHz)

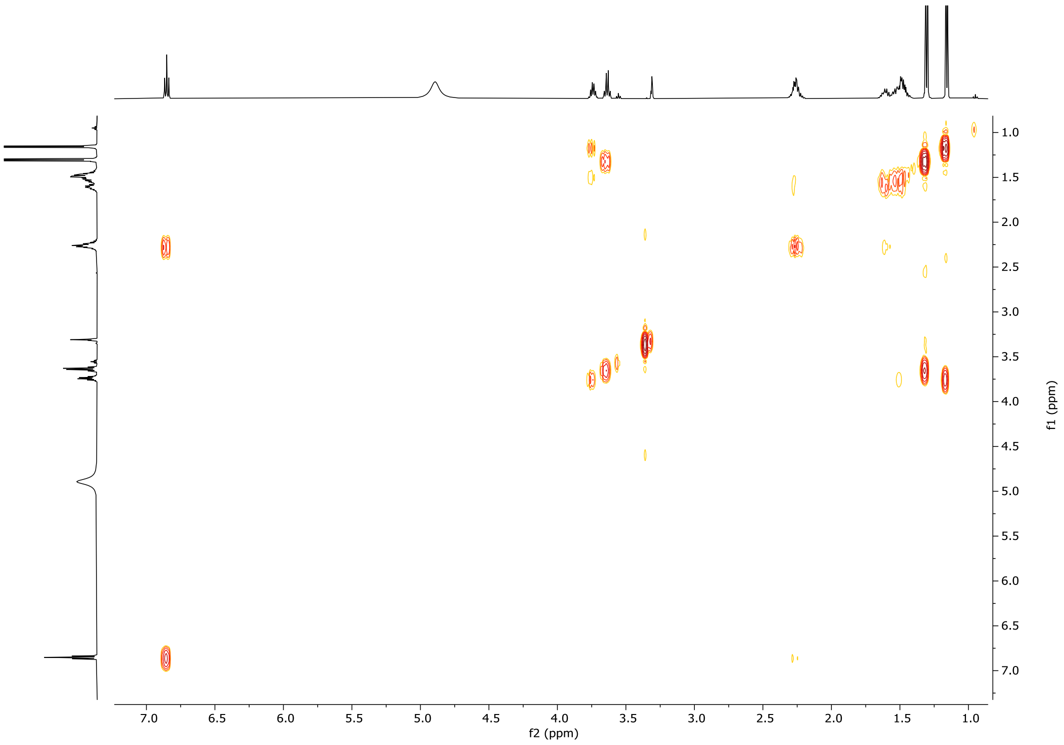

Figure S11. ^{1}H-^{1}H COSY spectrum of compound **2** (CD_3OD, 500 MHz)

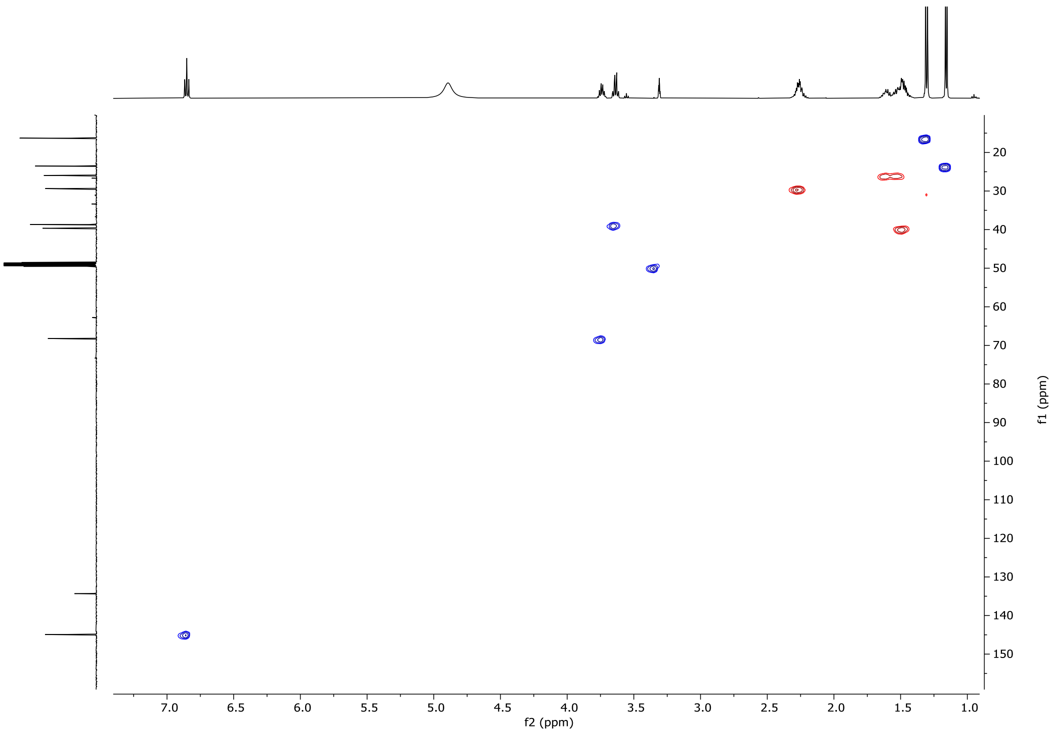

Figure S12. gHSQC spectrum of compound **2** (CD_3OD, 500 MHz)

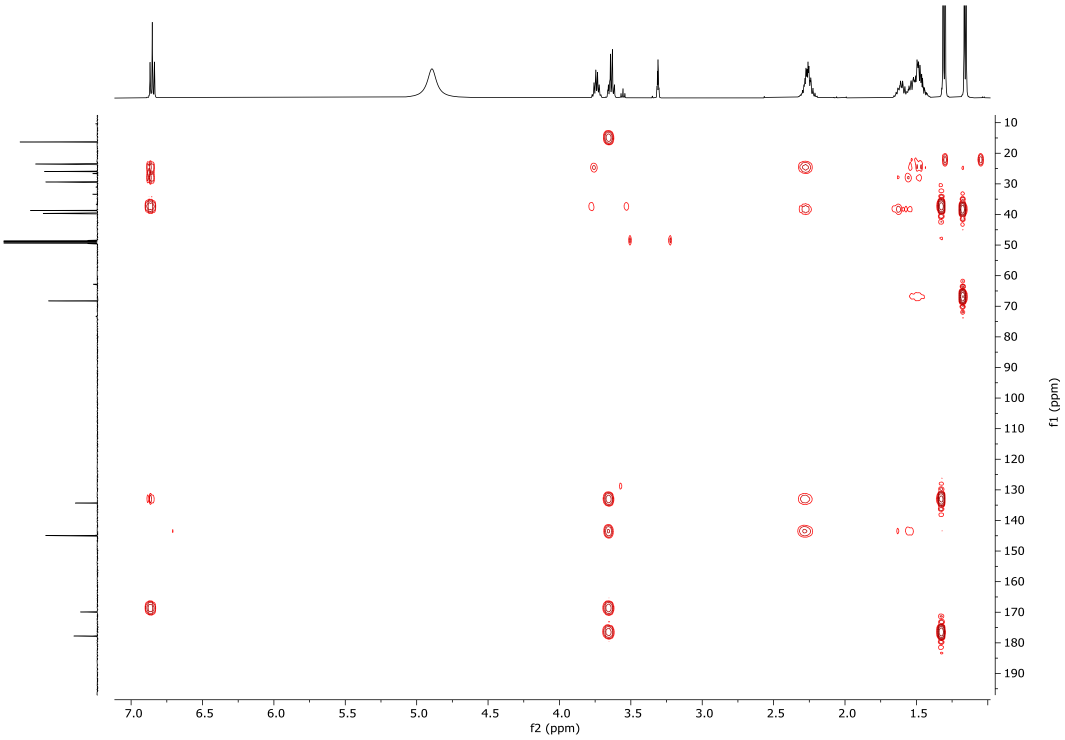

Figure S13. HMBC spectrum of compound **2** (CD_3OD, 500 MHz)

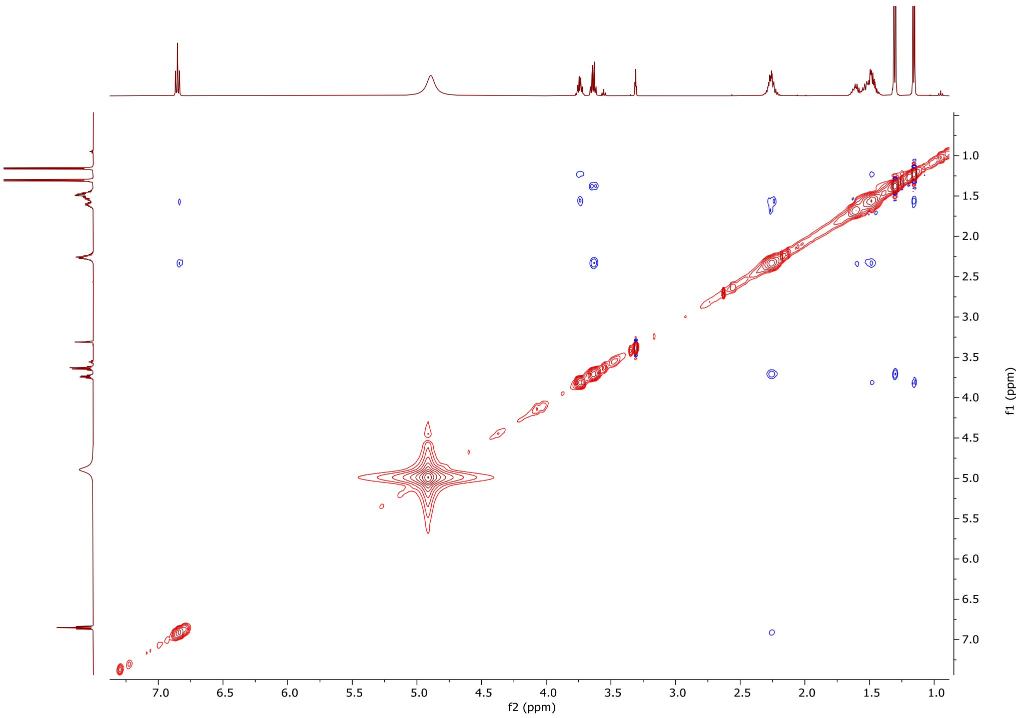

Figure S14. NOESY spectrum of compound **2** (CD_3OD, 500 MHz)

Elemental Composition Report **Page 1**

Tolerance = 5.0 PPM / DBE: min = -1.5, max = 100.0
Element prediction: Off
Number of isotope peaks used for i-FIT = 3

Monoisotopic Mass, Even Electron Ions
892 formula(e) evaluated with 5 results within limits (up to 50 best isotopic matches for each mass)
Elements Used:
C: 0-58 H: 0-70 N: 0-8 O: 0-11 Na: 0-1 S: 0-1
Carmen E
ESI (22-069) Carmen E (HYP6C7K 38-42) 52 (2.227) 1: TOF MS ES+ 6.42e+003

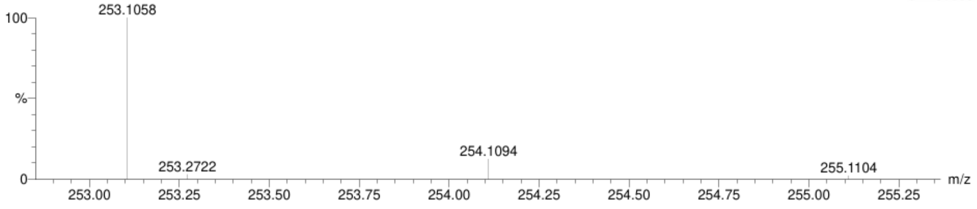

	Mass	RA	Calc. Mass	mDa	PPM	DBE	i-FIT	i-FIT (Norm)	Formula
Minimum:		30.00				-1.5			
Maximum:		100.00		5.0	5.0	100.0			
	253.1058	100.00	253.1052	0.6	2.4	2.5	16.2	0.0	C11 H18 O5 Na
			253.1049	0.9	3.6	6.5	21.1	4.9	C9 H13 N6 O3
			253.1065	-0.7	-2.8	7.5	21.9	5.7	C12 H14 N4 O Na

Figure S15. HRESIMS of compound **2**

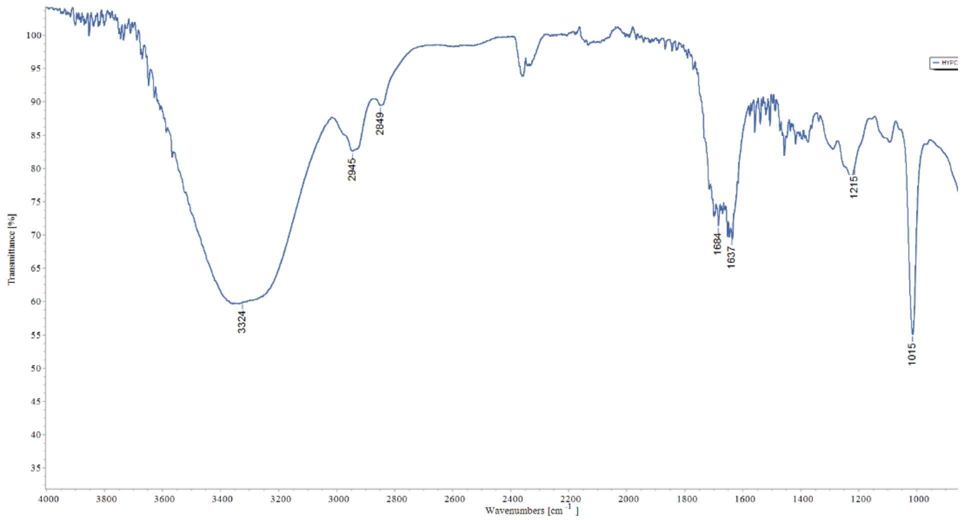

Figure S16. IR spectrum of compound **2**

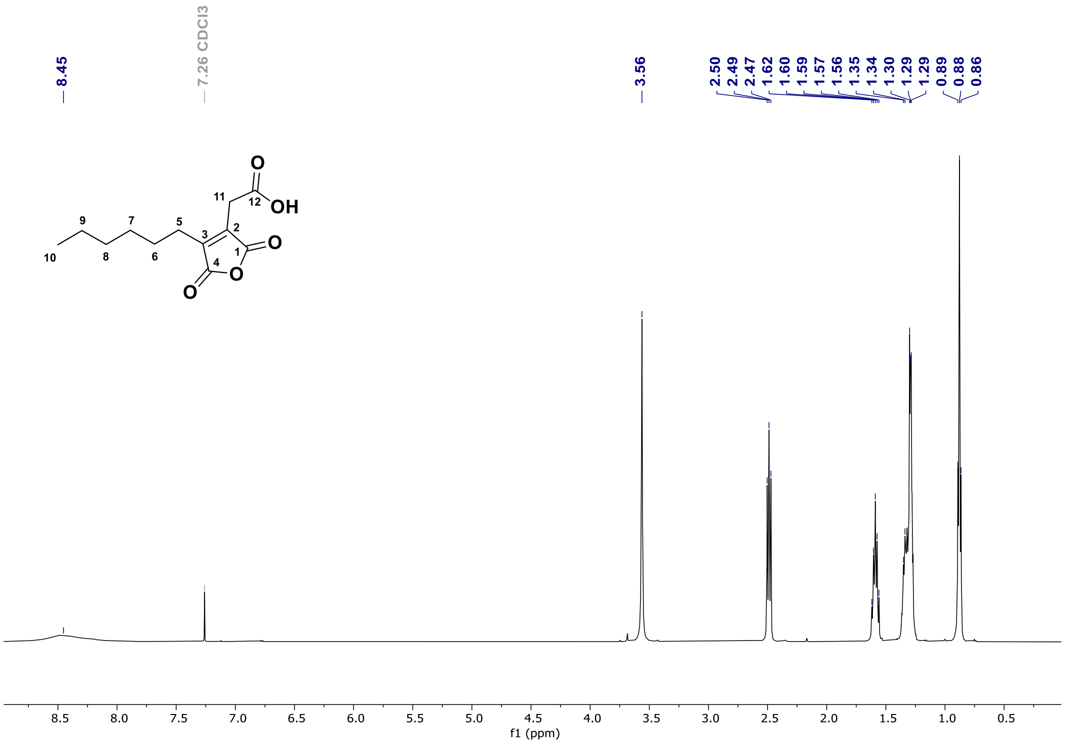

Figure S17. ^{1}H-NMR spectrum of compound **4** ($CDCl_3$, 500 MHz)

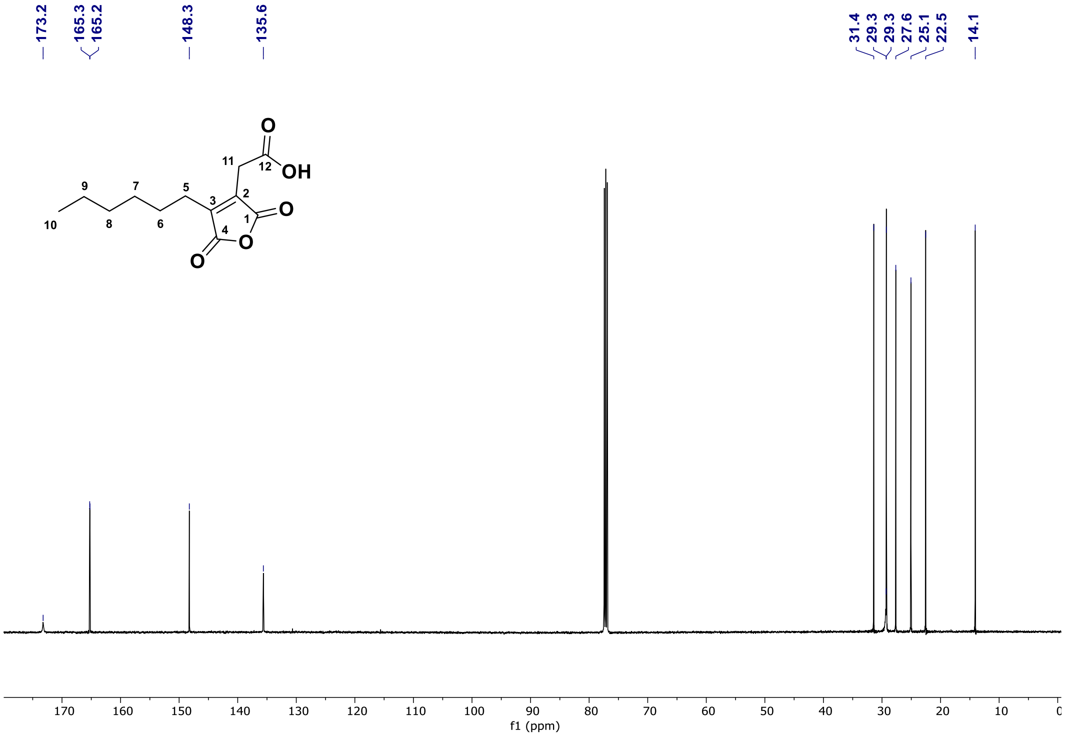

Figure S18. ^{13}C-NMR spectrum of compound **4** ($CDCl_3$, 125 MHz)

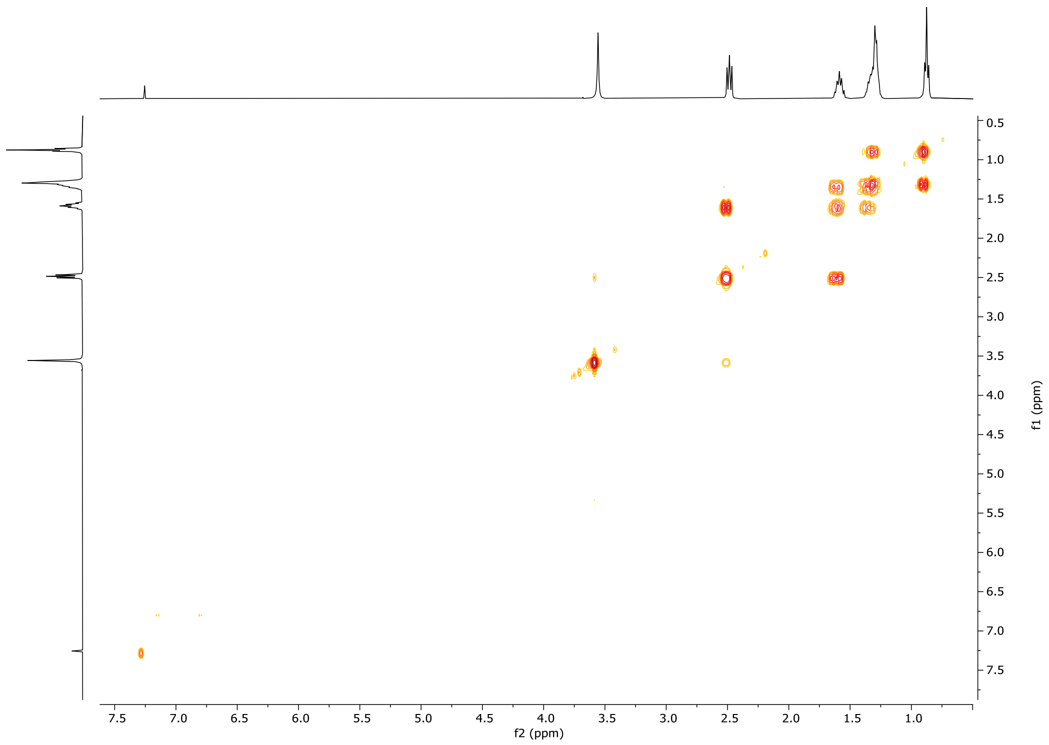

Figure S19. ^{1}H-^{1}H COSY spectrum of compound **4** ($CDCl_3$, 500 MHz)

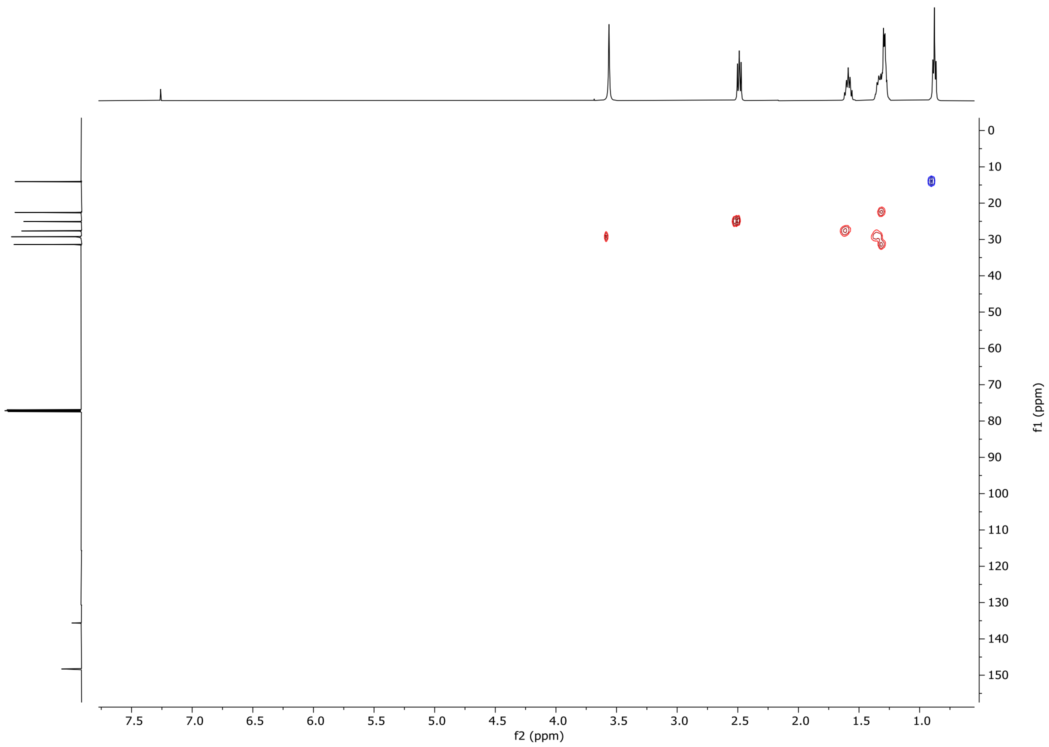

Figure S20. gHSQC spectrum of compound **4** ($CDCl_3$, 500 MHz)

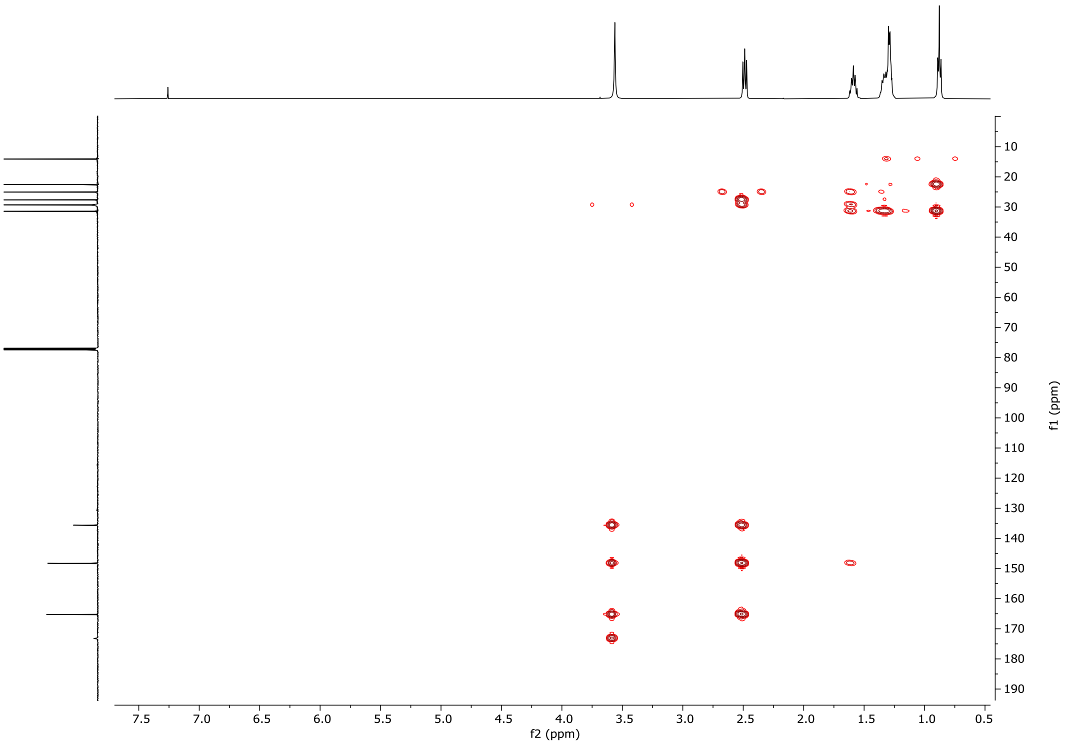

Figure S21. HMBC spectrum of compound **4** ($CDCl_3$, 500 MHz)

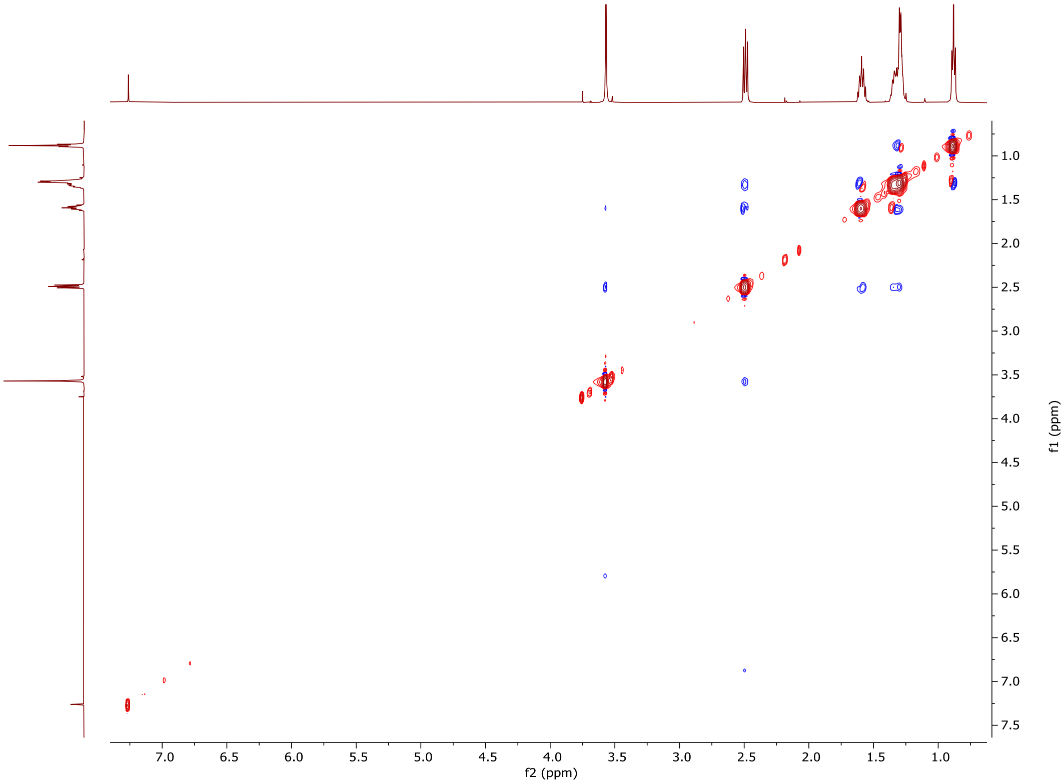

Figure S22. NOESY spectrum of compound **4** ($CDCl_3$, 500 MHz)

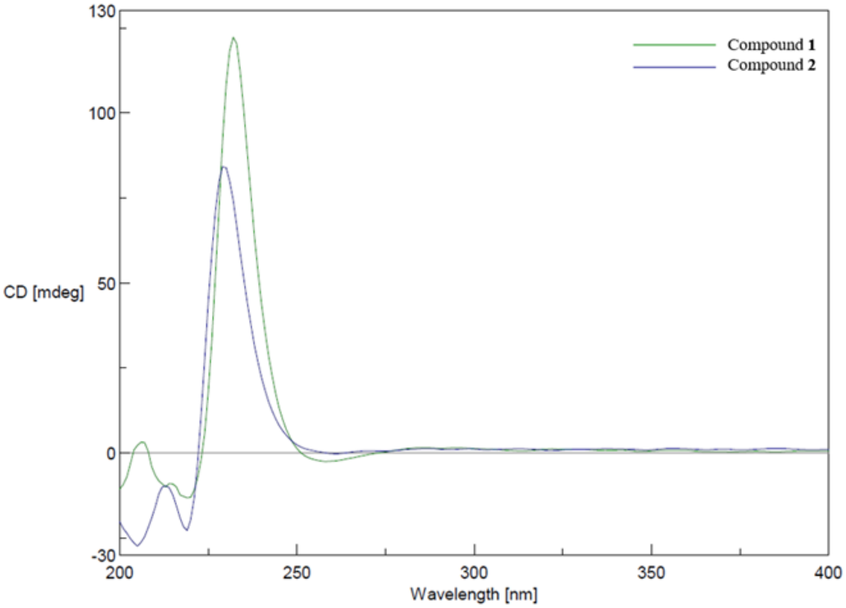

Figure S23. Experimental ECD spectra of compounds **1**–**2**

Table 1. ^{1}H NMR (500 MHz) and ^{13}C NMR (125 MHz) data for **4** in $CDCl_3$.

Position	δ_H, mult (*J* in Hz)	δ_C, type
**1**		165.3 [a], C
**2**	3.63, q (7.1)	135.6, C
**3**		148.3, C
**4**		165.2 [a], C
**5**	2.49 t, (7.7)	25.1, CH_2
**6**	1.59, quint (7.5)	27.6, CH_2
**7**	1.30, m	29.3, CH_2
**8**	1.30, m	31.4, CH_2
**9**	1.30, m	22.5, CH_2
**10**	δ 0.88, t, (6.7)	14.1, CH_3
**11**	3.56 s	29.3, CH_2
**12**		173.2, C

[a] These carbon can be interchanged.

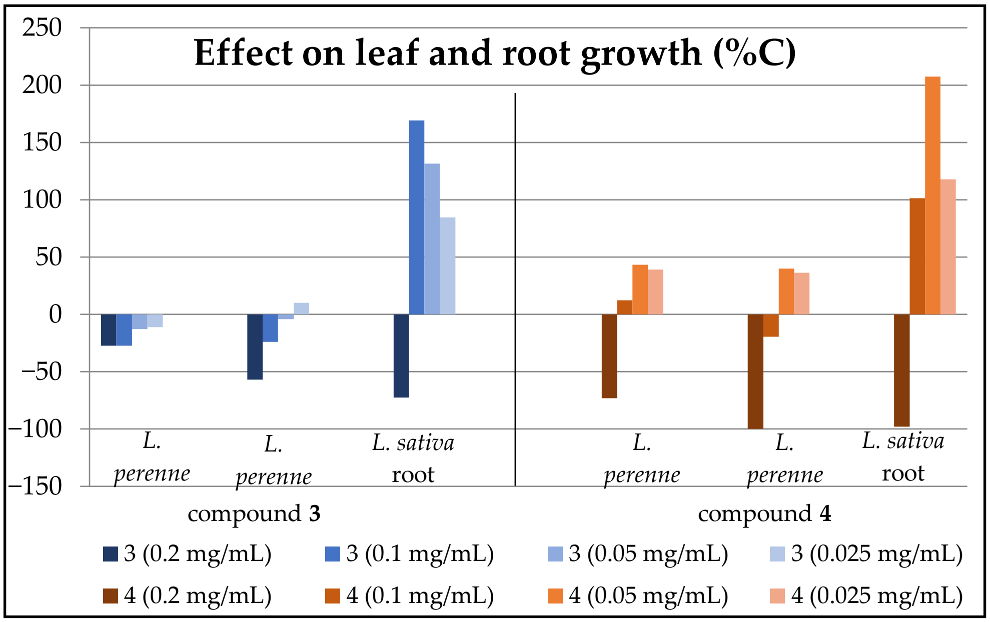

Figure S24. Phytotoxic effects of compounds **3** and **4** on *L. perenne* leaf and root growth and *L. sativa* root growth in lower doses (doses tested at 0.2, 0.1, 0.05 and 0.025 mg/mL)
